# Geriatric assessment and the variance of treatment recommendations in geriatric patients with gastrointestinal cancer—a study in AIO oncologists

**DOI:** 10.1016/j.esmoop.2022.100761

**Published:** 2023-01-11

**Authors:** M. Büttelmann, R.D. Hofheinz, A. Kröcher, U. Ubbelohde, S. Stintzing, A. Reinacher-Schick, M. Bornhäuser, G. Folprecht

**Affiliations:** 1 TU Dresden / University Hospital Carl Gustav Carus, National Center for Tumor Diseases (NCT/UCC), Medical Dept. I, Dresden, Germany; 2 University Hospital Mannheim, Mannheim, Germany; 3 Charité - Universitaetsmedizin Berlin, Department of Hematology, Oncology, and Cancer Immunology (CCM), Berlin, Germany; 4 Ruhr University Bochum, St. Josef Hospital, Department of Hematology, Oncology and Palliative Care, Bochum, Germany

**Keywords:** geriatric assessment, chemotherapy, geriatric oncology, survey

## Abstract

**Background:**

Geriatric assessment (GA) is recommended to detect vulnerabilities for elderly cancer patients. To assess whether results of GA actually influence the treatment recommendations, we conducted a case vignette-based study in medical oncologists.

**Materials and methods:**

Seventy oncologists gave their medical treatment recommendations for a maximum of 4 out of 10 gastrointestinal cancer patients in three steps: (i) based on tumor findings alone to simulate the guideline recommendation for a ‘50-year-old standard patient without comorbidities’; (ii) for the same situation in elderly patients (median age 77.5 years) according to the comorbidities, laboratory values and a short video simulating the clinical consultation; and (iii) after the results of a full GA including interpretation aid [Barthel Index, Cumulative Illness Rating Scale (CIRS), Geriatric 8 (G8), Geriatric Depression Scale (GDS), Mini Mental Status Examination (MMSE), Mini-Nutritional Assessment (MNA), Timed Get Up and Go (TGUG), European Organisation for Research and Treatment of Cancer Quality of Life Questionnaire-30 (EORTC QLQ-C30), stair climb test].

**Results:**

Data on 164 treatment recommendations were analyzed. The recommendations had a significantly higher variance for elderly patients than for ‘standard’ patients (944 versus 602, *P* < 0.0001) indicating a lower agreement between oncologists. Knowledge on GA had marginal influence on the treatment recommendation or its variance (944 versus 940, *P* = 0.92). There was no statistically significant influence of the working place or the years of experience in oncology on the variance of recommendations. The geriatric tools were rated approximately two times higher as being ‘meaningful’ (53%) and ‘useful for the presented cases’ (49%) than they were ‘used in clinical practice’ (19%). The most commonly used geriatric tool in patient care was the MNA (30%).

**Conclusions:**

The higher variance of treatment recommendations indicates that it is less likely for elderly patients to get the optimal recommendation. Although the proposed therapeutic regimen varied higher in elderly patients and the oncologists rated the GA results as ‘useful’, the GA results did not influence the individual recommendations or its variance. Continuing education on GA and research on implementation into clinical practice are needed.

## Highlights

• Oncologists have a lower agreement (higher variance) when recommending treatments for elderly compared to younger patients.
• GA results had no influence on the treatment recommendations or on the variance of the recommendations.
• Working experience as oncologist and working place had no significant influence on the variance.

## Introduction

The frequency of malignant tumors increases with age. The median age of onset was ∼70 years in 2018 in Germany.^1^ Similarly, it has been projected that by 2030 70% of all cancers will be diagnosed in older adults in the United States.^2^ While the burden of malignant diseases in the group of elderly persons is significantly higher than in their younger counterparts, evidence-based data on the optimal treatment regimen are scarce. One of the main reasons for this lack of hard data is that most clinical trials are conducted in a population that is younger than that encountered in the real world.^3^ This phenomenon has been known for more than two decades.^4^, ^5^, ^6^ However, there are attempts and appeals from oncological societies such as the American Society of Clinical Oncology (ASCO), European Society of Medical Oncology (ESMO), European Organisation for Research and Treatment of Cancer (EORTC), International Society of Geriatric Oncology (SIOG) as well as state actors such as the National Institutes of Health (NIH) to promote the inclusion of older patients in larger trials or designing trials especially for this patient group.^7^, ^8^, ^9^, ^10^, ^11^

Aging is a very heterogeneous process and may lead to inter-individual differences and impairments^12^^,^^13^ which may or may not be clinically apparent. While the association between the age by itself and an unfavorable outcome is unclear, the link between a decreased functional status and a poorer outcome has been well documented.^14^, ^15^, ^16^, ^17^ A strict cut-off from which age a patient is to be considered ‘old’ is not known. Generally, it is assumed that from the age of 65 years, patients have a higher risk of serious deficiencies, and therefore, a geriatric assessment (GA) is recommended for those patients.^8^^,^^18^^,^^19^ A GA consists of various validated tools that aid to identify areas that are typically impaired in older patients.^20^, ^21^, ^22^ While in geriatric departments a GA is carried out regularly, it is rarely used in elderly oncological patients.^23^^,^^24^ Limitations in supporting staff, in training or knowledge about GA or lack of time are regarded as the most common barriers.^23^ To address the issues of a lack of time and support staff, one possible strategy is to first carry out a screening which may be followed up by a full GA if potential problems are detected.^25^ If carried out correctly, a GA may influence the choice or intensity of therapy^19^^,^^24^, ^25^, ^26^, ^27^, ^28^ as well as predict toxicity, completion of therapy and mortality.^28^, ^29^, ^30^, ^31^, ^32^, ^33^, ^34^ However, there are also contradictory data from studies where primary endpoints such as prediction of toxicity, hospitalization, quality of life, completion of therapy, progression-free and overall survival were not significantly improved by a GA.^35^, ^36^, ^37^ It is a problem in clinical practice that the information generated by a GA is not always used or acted upon.^26^^,^^36^ This may also be due to a lack of training or research results. More data on the clinical ramification of GA are needed.

To investigate the treatment recommendation in elderly patients and the contribution of a GA, we asked medical oncologists to participate in a study on treatment recommendations for gastrointestinal (GI) cancer patients and to give their treatment recommendations according to tumor findings (‘tumor board of an otherwise healthy, younger person’), after having seen a video sequence of the patient (simulating the situation of a clinical consultation) and after the GA results had been disclosed. Information on demographics of participating oncologists and practice characteristics was collected.

## Materials and methods

### Survey development and deployment

Patients with GI tumors were recruited for this study at University Hospital Carl Gustav Carus, Dresden, Germany between September 2018 and August 2019. After a written informed consent was obtained, a GA with commonly used screening instruments [Barthel Index (BI), Cumulative Illness Rating Scale (CIRS), Geriatric 8 (G8), Geriatric Depression Scale (GDS), Mini Mental Status Examination (MMSE), Mini-Nutritional Assessment (MNA), Timed Get Up and Go (TGUG), EORTC Quality of Life Questionnaire-C30 (QLQ-C30), reviewed by SIOG^22^] was carried out as well as a stair climb test (SCT). Results of interventions based on this geriatric screening were not part of the study. A video of the patient walking into the consultation room including a short dialog to sum up their medical history was recorded and the video sequences were shortened to ∼2 min. The 10 most complex patient cases were selected to be presented in a survey to German-speaking oncologists at an oncological convention in Dresden/Radebeul, at the annual AIO meeting, Berlin and in a web-based survey in AIO members between September 2019 and March 2020. Each patient was presented to the oncologists in three steps. The time to complete the survey showing two different patients was ∼15-20 min.

**Figure 1:**
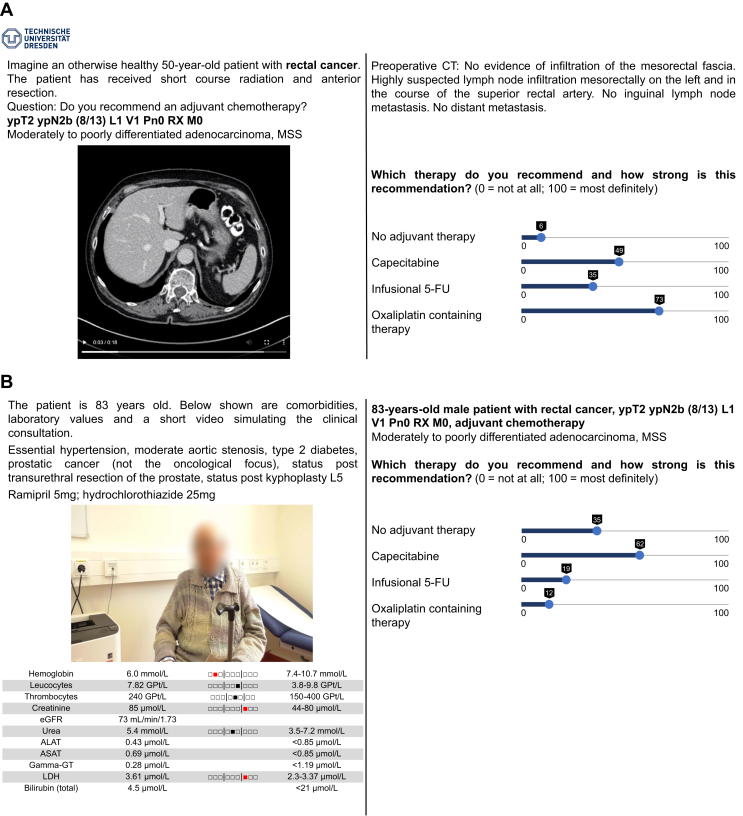

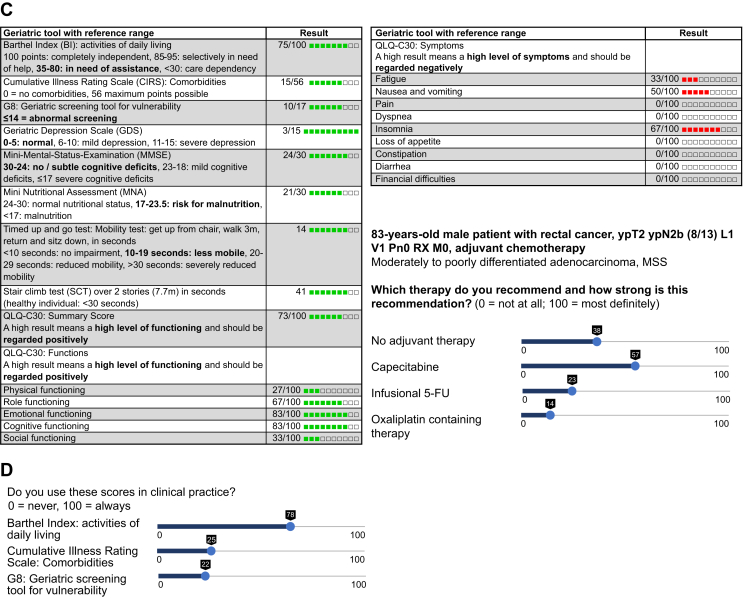
**Part of the survey to the oncologists (translated from German)**. (A) Simulation of tumor board situation: 50-year-old patient, cancer stage, histology, grading, immunohistochemistry, molecular biology, relevant imaging and no additional comorbidities. (B) Simulation of a clinical consultation of an elderly patient showing age, comorbidities, medication, examinations (e.g. endoscopy), laboratory values and video of the patient history. (C) Simulation of a situation of optimized care showing the results of the GA, normal values shown for reference. (D) Additional questions: Do you use these [geriatric] scores in clinical practice? GA, geriatric assessment.

In the first step, a tumor board situation was simulated and should result in a guideline-based treatment recommendation for younger patients without comorbidities. For that purpose, the participants were asked to imagine a 50-year-old patient for whom the cancer stage, histology, grading, immunohistochemistry, molecular biology, examinations (e.g. endoscopy) and relevant imaging were presented. Participants were asked to recommend or advise against several options of therapeutic regimens using a slider on a range of 0-100, similar to a visual analog scale (Figure 1A).

In the second step, a clinical consultation of an elderly patient with the same tumor was simulated. In addition to the information already known from step 1, the actual patient age, comorbidities, medication and laboratory values were shown together with the video of the patient simulating a consultation situation. Participants were asked to make a treatment recommendation on the same scales as step 1 (Figure 1B).

In the third step, the results of the GA were disclosed. The normal values of each instrument and a short interpretation aid were provided to the participants for reference. This step was used to simulate a setting of optimized care for elderly patients including the GA. Finally, participants were asked to make a treatment recommendation again (Figure 1C).

Before viewing the case stories, participants were asked their opinion regarding the geriatric tools used in the survey, their use in clinical practice and whether the instruments were regarded as meaningful in general (Figure 1D). After completing two case vignettes, participants were asked to rate the same tools with regard to the usefulness in the choice of therapy for the two patients. An option to vote in two additional patients was given.

Furthermore, the specialization of the participants, years of experience and place of work (hospital, outpatient unit, private oncologists) were part of the questionnaire.

### Data analysis

A descriptive statistical analysis was conducted for the responses to survey questions such as the participants’ background, rating of geriatric tools and recommendation of therapeutic regimens. Data visualization was carried out via column charts and radar plots. In the radar plot, the mean and the standard deviation for each single recommendation (i.e. no chemotherapy, regimen 1, regimen 2, radio-chemotherapy) are printed in the different axes per patient.

To analyze the agreement between the treatment recommendations, we calculated the variance (squared standard deviation of each recommendation).

Differences were considered significant if *P* < 0.05 without correction for multiple testing.

### Ethics

All patients had given an informed consent to use their data and videos in the survey. The responsible ethics committee at the Technical University Dresden approved the study before initiation.

## Results

### Patients

The median age of the 10 patients featured in the vignettes was 77.5 years (range 73-84 years). Eight patients were male and two were female. Patients’ malignancies included adenocarcinoma of the gastroesophageal junction (4), pancreatic (3), colorectal (2) and gastric cancers (1). The treatment situation was neoadjuvant (5), adjuvant (4) and palliative (3).

### Oncologists

Of the 76 participants, 6 were excluded due to their non-appropriate specialization (surgery, gynecology, neuro-oncology, pneumology and others). The majority were board-certified specialists (91%) with working experience as specialists of >6 years (80%). Most participants were hemato-/oncologists. Regarding the place of work, 33% of participants worked in a hospital ward, 33% in hospital-based outpatient units and 29% in a private medical office. Out of the hospital-based participants, most worked in larger hospitals (>800 beds, 81%) and were employed as senior physicians (60%, Table 1).

**Table 1:** Participants’ demographics

Resident, *n* (%)	5 (7)
Experience in years as resident	
0 to <2	1 (20)
≥2 to <4	4 (80)
≥4 to 6	0
≥6	0
Specialist, *n* (%)	64 (91)
Experience in years as specialist	
0 to <2	4 (6)
≥2 to <4	4 (6)
≥4 to 6	5 (8)
≥6	51 (80)
Position within clinic, *n* (%)	
Specialist	6 (14)
Senior physician	26 (60)
Chief physician	11 (26)
Others (1× palliative care)	11 (2)
Area of focus, *n* (%)	
Hematology-oncology	58 (83)
Gastroenterology	7 (10)
Internal medicine	2 (3)
Palliative care	2 (3)
Others (= gastrointestinal oncology)	1 (1)
Place of work, *n* (%)	
Ward	23 (33)
Oncology day care unit or outpatient clinic of a hospital	23 (33)
Ambulant, e.g. medical center, solo or group practice	20 (29)
No direct patient contact	2 (3)
Others (= 1× medical center and hospital, 1× endoscopy)	2 (3)
Number of beds (only for participants working in a hospital), *n* = 43, *n* (%)	
<400	0
400-800	8 (19)
>800	35 (81)

Participants, *n* = 70.

The participants rated the geriatric scores on a visual analog scale. The mean for using the geriatric scores in clinical practice was 19.4 (±28.4). The mean for regarding the scores as generally meaningful was 52.5 (±32.2), and the mean for the question whether the GA was helpful in deciding a therapeutic regimen for the vignettes was 48.7 (±30.5). The differences were significant (*P* < 0.0001, Table 2).

**Table 2:** Rating of the geriatric tools used and use in clinical practice

Rating of geriatric tools	Mean (%) ± SD	Level of significance		*n*
‘Use in clinical practice’	19.4 ± 28.4	<0.0001		70
‘Meaningful’	52.5 ± 32.2		<0.0001	70
‘Useful'	48.7 ± 30.5			67
**Rating of the single instruments**				
Barthel Index				
‘Use in clinical practice’	27.9 ± 32.1	<0.0001		70
‘Meaningful’	58.0 ± 29.0		0.2704	70
‘Useful’	54.2 ± 30.4			67
Cumulative Illness Rating Scale				
‘Use in clinical practice’	17.7 ± 28.3	<0.0001		69
‘Meaningful’	52.8 ± 30.9		0.3475	69
‘Useful’	50.2 ± 30.8			66
Geriatric 8				
‘Use in clinical practice’	15.9 ± 28.0	<0.0001		70
‘Meaningful’	46.6 ± 34.5		0.9074	70
‘Useful’	47.9 ± 30.2			67
Geriatric Depression Scale				
‘Use in clinical practice’	11.3 ± 21.5	<0.0001		70
‘Meaningful’	41.1 ± 29.6		0.0861	70
‘Useful’	35.4 ± 28.9			67
Mini Mental Status Examination				
‘Use in clinical practice’	26.1 ± 28.9	<0.0001		70
‘Meaningful’	62.3 ± 28.9		0.0051	70
‘Useful’	51.6 ± 30.7			67
Mini-Nutritional Assessment				
‘Use in clinical practice’	29.5 ± 34.5	<0.0001		70
‘Meaningful’	63.0 ± 31.4		0.0332	70
‘Useful’	55.1 ± 30.1			67
Timed Get Up and Go				
‘Use in clinical practice’	21.5 ± 29.1	<0.0001		70
‘Meaningful’	58.3 ± 31.5		0.7406	70
‘Useful’	58.4 ± 27.6			67
Stair climb test				
‘Use in clinical practice’	27.2 ± 32.5	<0.0001		70
‘Meaningful’	63.5 ± 30.3		0.0055	70
‘Useful’	52.4 ± 30.1			67
Quality of Life Questionnaire-C30: summary score				
‘Use in clinical practice’	11.1 ± 19.7	<0.0001		70
‘Meaningful’	44.7 ± 32.4		0.6930	70
‘Useful’	44.0 ± 29.5			67
EORTC QLQ-C30: functions				
‘Use in clinical practice’	11.3 ± 20.0	<0.0001		70
‘Meaningful’	43.3 ± 32.3		0.7964	70
‘Useful’	43.1 ± 30.1			67
EORTC QLQ-C30: symptoms				
‘Use in clinical practice’	14.4 ± 25.4	<0.0001		70
‘Meaningful’	43.4 ± 32.8		0.8581	70
‘Useful’	43.5 ± 31.7			67

Use in clinical practice = ‘Do you use these [geriatric] tools in clinical practice?’; meaningful = ‘Do you consider these [geriatric] tools meaningful?’; useful = ‘Did you find these [geriatric] tools useful when making a decision on the therapeutic regimen in the vignettes shown?’.

Level of significance *P* < 0.05.

*n*, number of participants; EORTC QLQ, EORTC Quality of Life Questionnaire; SD, standard deviation.

The same trend was also observed for the results of the individual geriatric tools. ‘Use in clinical practice’ was always significantly lower than the ratings as ‘meaningful’ or ‘helpful in the presented case’. Most of the geriatric tools were regarded as being slightly less useful in the case vignettes than they were regarded to be meaningful in general. These differences were only significant for MMSE, MNA and SCT. The TGUG and EORTC QLQ-C30 symptom scale were rated to be slightly more useful than meaningful, although these differences were not significant (Table 2).

As described in the Materials and methods section, the patient vignettes were presented in three steps. Data from 70 participants who had given a total of 164 recommendations were analyzed.

**Figure 2:**
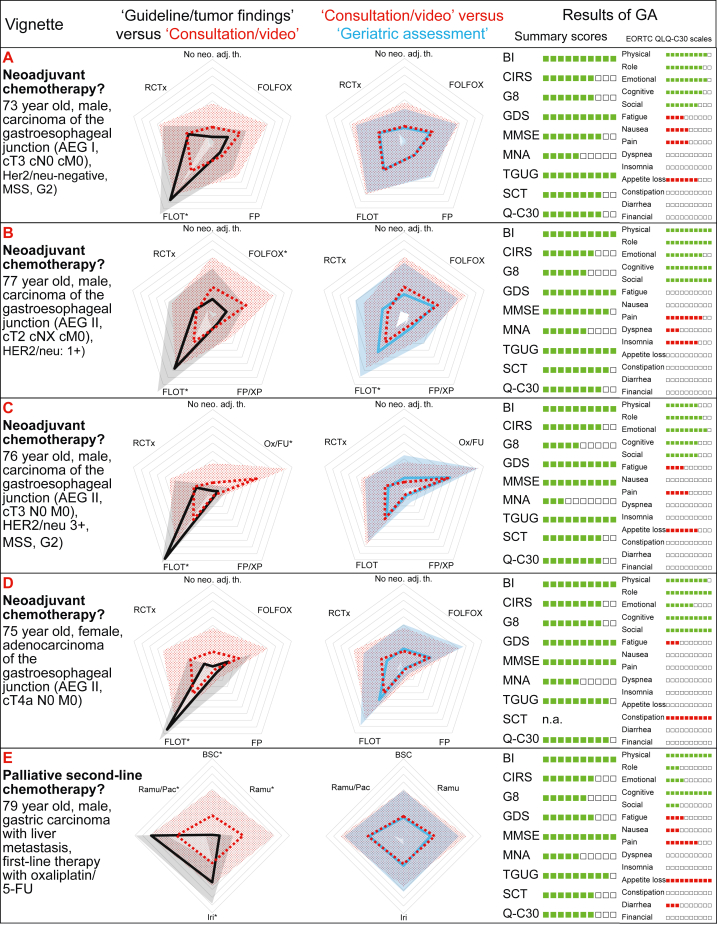

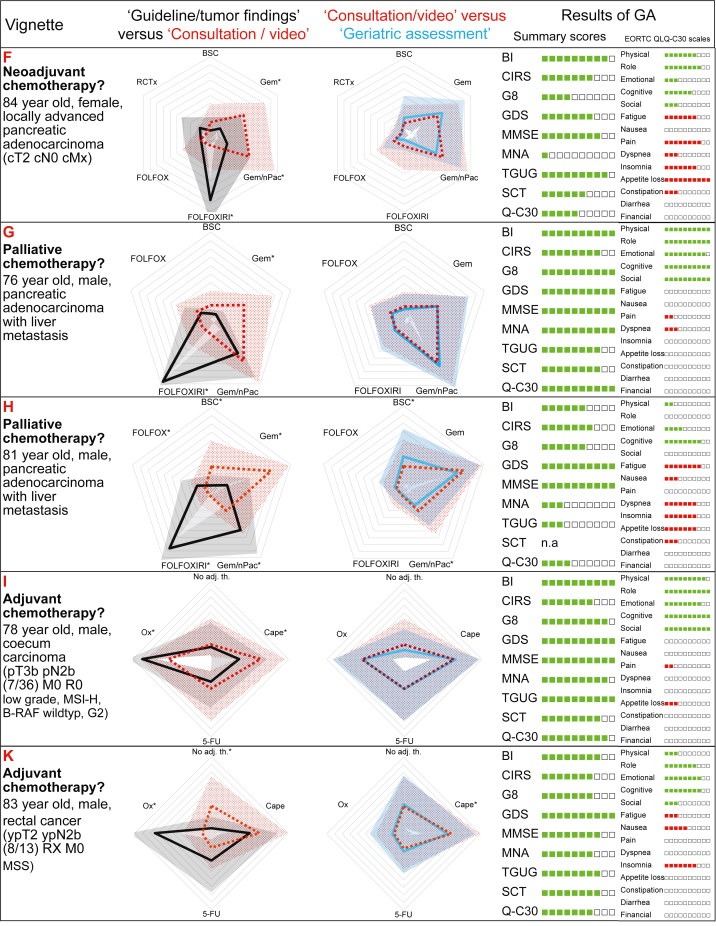
**Combined radar plots of recommended therapeutic regimens**. The black, red and blue lines show the median, the gray, light red and light blue areas the standard deviations of the treatment recommendations according to the ‘guideline/tumor findings’ (‘please assume 50-year-old patient without comorbidities’, cross-sectional imaging, stage of disease provided; black graph), according to ‘consultation/video’ (actual age, video, comorbidities, medication, laboratory results provided; red graph) and with the additional results of the ‘geriatric assessment’ [including Barthel Index (BI), Cumulative Illness Rating Scale (CIRS), Geriatric 8 (G8), Geriatric Depression Scale (GDS), Mini Mental Status Examination (MMSE), Mini-Nutritional Assessment (MNA), Timed Get Up and Go (TGUG), EORTC Quality of Life Questionnaire-C30 (QLQ-C30), EORTC QLQ-C30 summary score (Q-C30), EORTC QLQ-C30 functioning scales and symptom scales (EORTC QLQ-C30 scales); blue graph). The gray lines delineate 10% steps, with significant differences between recommendations marked with an asterisk.

Figure 2 shows a combined radar plot of the recommended therapeutic regimens. In the first graph, step 1 (50 years old, no comorbidities, cross-sectional imaging, stage of disease) and step 2 (actual age, video, comorbidities, medication, laboratory results) are shown. In the second graph, steps 2 and 3 (elderly patients without or with results of GA) are shown. The individual data for all patient cases are provided in the Supplementary Material (Vignettes A-K), available at https://doi.org/10.1016/j.esmoop.2022.100761.

As visualized in the overview graph (Figure 2), large differences can be observed between step 1 and step 2 for the individual therapeutic options of a given patient. These differences were significantly different in 24 out of 48 therapeutic options. Between steps 2 and 3, the differences were less pronounced and significant in 4 out of 48 therapeutic options only.

As a parameter of the agreement between the recommendations of therapeutic regimens, we analyzed the variance of the treatment recommendations for each option. This variance was significantly smaller in step 1 than in step 2 expressing a higher agreement in the standard situation than in elderly patients [mean of variances 602 (step 1) versus 944 (step 2), *P* < 0.0001]. The variance of step 3 (940) was only slightly lower than in step 2 (*P* = 0.92) indicating that the agreement between the recommendations was not higher with known results of GA.

To investigate whether GA results had a higher impact in decision making for more frail patients, we additionally divided the cases according to the result of the GA into two groups (upper and lower half of results). This stratified analysis showed no consistent trends in the change of variance (data not shown).

**Figure 3:**
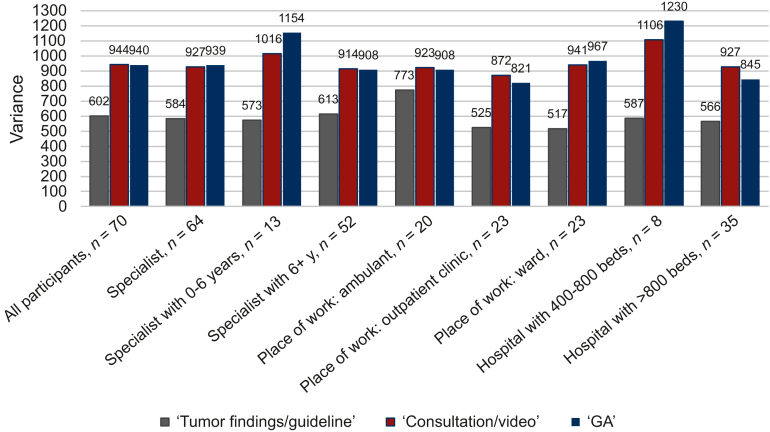
**Variance of recommendation according to subgroups of participants.** Ambulant = private medical office; specialist = board-certified specialist; residents (n = 5) not shown. ‘Guideline/tumor findings’ = please assume 50-year-old patient without comorbidities, cross-sectional imaging, stage of disease provided; consultation/video = actual age, video, comorbidities, medication, laboratory results provided; GA, geriatric assessment = results of Barthel Index (BI), Cumulative Illness Rating Scale (CIRS), Geriatric 8 (G8), Geriatric Depression Scale (GDS), Mini Mental Status Examination (MMSE), Mini-Nutritional Assessment (MNA), Timed Get Up and Go (TGUG), EORTC QLQ-C30 summary score (Q-C30), EORTC QLQ-C30 functioning scales and symptom scales (EORTC QLQ-C30 scales).

Furthermore, a subgroup analysis according to the demographics of participants was carried out. In step 1, participants working as private oncologists had a higher variance than participants based in a hospital. In step 2 and step 3, participants working in larger hospitals (>800 beds) and specialists with >6 years of experience showed a slightly lower variance than participants in smaller hospitals or specialists with <6 years of experience. However, these differences were not statistically significant (Figure 3).

## Discussion

In this study for geriatric patients with GI tumors and comorbidities, we found—not surprisingly—different treatment recommendations for elderly versus younger patients. However, the variance in the treatment recommendation in elderly patients is substantial and did not differ significantly after the disclosure of GA results. By providing all physicians the same information on a patient, we could exclude that the wider range of health status in elderly patients^13^ is the background for the higher variance in treatment recommendations. Because not all treatment options can be considered equally ‘right’, we hypothesize that it is substantially less likely for elderly patients to be treated with the optimal regimen. We had expected that the additional information of a GA would lead—by providing more standardized information—to a more homogenous treatment recommendation and to a reduced variability, but this was clearly not observed.

Interestingly, the variance in treatment recommendation was not markedly decreased even in more experienced oncologists which one might have expected. Other subgroup analyses by demographics and working place showed small differences in the variances between participant groups. However, these trends were neither significant nor consistent. Therefore, we hypothesize that it is not (much) more likely to receive the ‘right’ treatment recommendation from a more experienced physician or in a special setting. This is of concern and underlines that a more structured way of approaching elderly patients is urgently warranted—but contrasts with the fact that the GA results had finally very limited influence on the simulated treatment recommendation.

The variance in recommendation of treatment for a younger, 50-year-old patient without comorbidities (step 1) was not negligible, probably due to some controversies regarding the optimal treatment even for non-elderly patients, i.e. regarding the best neoadjuvant therapy for carcinoma of the gastroesophageal junction. However, the variance was significantly higher for older patients with chronic illnesses (944 versus 602, step 2 versus step 1). This is in line with the growing body of data that the variability in treatment recommendation is greater in geriatric patients than in younger individuals,^38^, ^39^, ^40^, ^41^, ^42^ but it expresses the uncertainty of the oncologists regarding the optimal treatment. While for younger patients without comorbidities a multitude of high-quality studies and guidelines are available leading to a stronger consensus on the choice of therapy, the increased variance for elderly patients with comorbidities might be driven by the lack of evidence-based data on the best treatment.^27^ The underrepresentation of elderly patients in clinical trials^4^, ^5^, ^6^ contributes to this problem. The highly selected elderly patients actually enrolled in the standard trials are limiting the generalizability of the results of subgroup analyses, and trials focusing on elderly patients are rare. As a result, the theoretical knowledge on the optimal treatment decision is limited for the elderly population.

Geriatric screening tools used in our survey are developed to identify vulnerabilities that might not be captured in the routine assessment.^18^^,^^19^ Two recently published randomized trials have demonstrated that a comprehensive intervention following GA decreased the toxicity^28^^,^^34^ without influence on the survival.^28^ In addition, there are instruments to estimate the chemotherapy risks like CARG^29^^,^^43^ or CRASH.^30^ Although these tools allow a better prediction of risks and have been proven to increase the tolerability of the treatment, disease-specific randomized trials for the best treatment option based on a standardized assessment are rare or lacking for most situations. This leaves the physician alone in the therapeutic dilemma to weigh the risks and benefit on adhering more to the general treatment guideline and to avoid undertreatment and to avoid unnecessary toxicity. Some strategies include dose reduction that has evidence, i.e. in palliative treatment of gastric and colon cancer,^44^, ^45^, ^46^ and adjusting the therapy based on the individual tolerability. While this strategy might be appropriate in palliative therapy, there are clear limitations for this strategy in perioperative or adjuvant settings.

The perception that the GA has limited answers to this question might be one explanation for the fact that geriatric instruments were not used regularly. Even the most popular tools MNA, BI and SCT (which is not part of the SIOG reviewed tests) were only used by 30%, 28% and 27% of participants, and because of the social desirability bias,^47^ these proportions might even be overestimated. This is in line with a recent survey among ASCO members reporting the use of formally validated tools by 29% of respondents.^23^ Despite the strong recommendation by medical associations to carry out a GA for potentially vulnerable elderly,^8^^,^^18^^,^^19^ the implementation into clinical practice is still lacking. To overcome a potential lack of experience with the results of the GA, an interpretation aid was given in our survey. It is promising, however, that even though not used regularly, the geriatric tools were regarded to be meaningful in general and helpful in the vignettes by roughly half of the participants. To lower the threshold of using a GA, visual aids like those used in our study may be beneficial.

There are also limitations to this study. The sample size of 70 participants might be regarded as relatively small but is related to the relatively long survey. With German as the language of communication, the survey was restricted to German speakers, potentially limiting the generalizability of the results. With a clear focus on geriatric patients, it is possible that participation was biased toward physicians with special interest in this geriatric oncology. There are also limitations in the study design. To avoid additional complexity of the treatment recommendation, only different protocols, but no additional option for dose reduction, were provided which may be applied in clinical practice for older or frail patients^48^^,^^49^ and have been successfully validated for some malignancies and stages.^44^, ^45^, ^46^ Furthermore, we had not provided the results of the CARG^29^ or CRASH^30^ tools that can be used to estimate the chemotherapy risks. While the format of vignettes has been validated to survey physicians^50^ and has already been successfully deployed in the geriatric oncology setting,^27^^,^^38^^,^^51^^,^^52^ vignettes remain a simulation. Hence, the results should be extrapolated carefully to clinical practice. The clear advantage of the survey situation is the standardization of the questions and the exclusion of patient factors as reasons for the variability.

In conclusion, the variance of recommended therapeutic regimens was significantly higher for elderly patients with comorbidities than for younger patients without comorbidities indicating a lower likelihood to receive the optimal treatment. The additional information of a GA did not influence the variance significantly. While a GA is recommended for elderly patients and can identify potential problems, it was not used routinely by most oncologists. Further efforts to promote GA and its implementation into clinical practice should include recommendations for daily clinical practice and in clinical trials to provide subgroup analyses based on a standardized evaluation by GA tools.
