# Supplementary material for: Geriatric assessment and the variance of treatment recommendations in geriatric patients with gastrointestinal cancer—a study in AIO oncologists
Source: ESMO Open. 2023 Jan 11;8(1):100761. doi: 10.1016/j.esmoop.2022.100761 (PMC10024156; doi:10.1016/j.esmoop.2022.100761)
Supplement: Supplementary materials [file mmc1.pdf]

Supplementary material: Patient cases

Vignette A

73-year-old male with a carcinoma of the gastroesophageal junction AEG I (cT3 cN0 cM0, poorly differentiated mucinous adenocarcinoma, Her2/neu-negative, MSS, G2).

Question: Neoadjuvant chemotherapy?

The following options were given:

- No neoadjuvant therapy (no neo adj. th.)
- Folinic acid, 5-fluorouracil and oxaliplatin (FOLFOX)
- 5-FU/cisplatin (FP)
- 5-fluorouracil, folinic acid, oxaliplatin and docetaxel (FLOT)
- Radiochemotherapy (RCTx)

Table S3 shows the descriptive statistics on the therapeutic recommendations followed by a graphical presentation. In the first graph, step 1 (50-years-old, no comorbidities, cross-sectional imaging, stage of disease) and step 2 (actual age, video, comorbidities, medication, lab results) are shown. In the second graph, step 2 and step 3 (results of GA) are shown.

Table S1: Recommended therapeutic regimes for vignette A

	No neo adj. th	FOLFOX	FP	FLOT	RCTx
Guideline / tumor findings					
Mean ± SD	6 ± 13	19 ± 24	16 ± 26	87 ± 21	30 ± 32
Variance	171	556	661	451	1030
„Consultation / video“					
Mean ± SD	18 ± 29	36 ± 34	21 ± 31	43 ± 36	33 ± 35
Variance	814	1143	951	1264	1209
„Geriatric assessment“					
Mean ± SD	16 ± 23	32 ± 34	21 ± 33	42 ± 35	32 ± 30
Variance	517	1158	1075	1193	885

“Guideline / tumor findings“: 50-years-old patient without comorbidities, cross-sectional imaging, stage of disease provided; “consultation / video”: actual age, video, comorbidities, medication, lab results provided; “geriatric assessment”: BI [Barthel Index], CIRS [Cumulative Illness Rating Scale], G8 [Geriatric 8], GDS [Geriatric Depression Scale], MMSE [Mini Mental Status Examination], MNA [Mini-Nutritional Assessment], TGUG [Timed Get Up and Go], QLQ-C30 [EORTC Quality of Life Questionnaire-C30]). FLOT = 5-fluorouracil, folinic acid, oxaliplatin and docetaxel, FOLFOX = folinic acid, 5-fluorouracil and oxaliplatin, FP = 5-FU/cisplatin, No neo adj. th. = no neoadjuvant therapy, RCTx = radiochemotherapy, n= 17

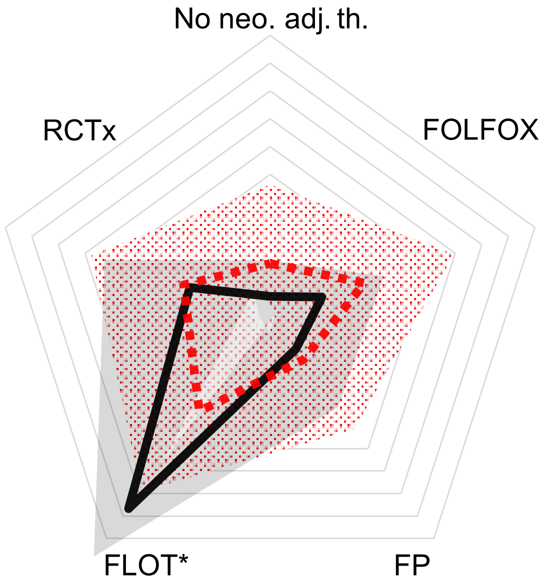

Figure S1: Radar plot for therapeutic recommendations for vignette A. The black and red lines show the median, the grey and light red areas the standard deviations of the treatment recommendations according to „guideline / tumor findings“ (“please assume 50-years-old patient without comorbidities”, cross-sectional imaging, stage of disease provided; black graph) and according to the “consultation / video” (actual age, video, comorbidities, medication, lab results provided; red graph); FLOT = 5-fluorouracil, folinic acid, oxaliplatin and docetaxel, FOLFOX = folinic acid, 5-fluorouracil and oxaliplatin, FP = 5-FU/cisplatin, No neo adj. th. = no neoadjuvant therapy, RCTx = radiochemotherapy, significant differences between recommendations marked with an asterisk, n= 17

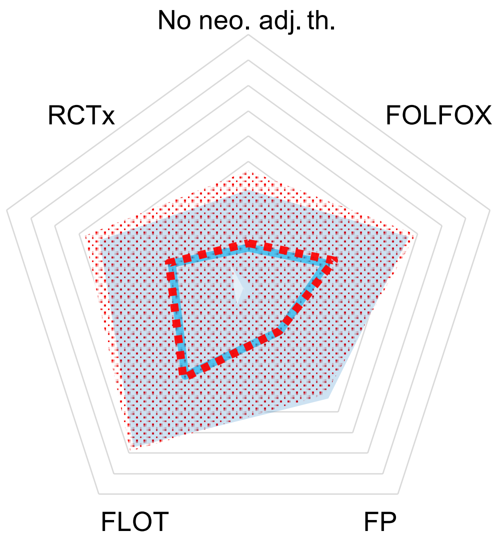

Figure S2: Radar plot for therapeutic recommendations for vignette A. The red and blue lines show the median, the light red and light blue areas the standard deviations of the treatment recommendations according "consultation / video" (actual age, video, comorbidities, medication, lab results provided; red graph) and according to the additional results of the "geriatric assessment" (including BI [Barthel Index], CIRS [Cumulative Illness Rating Scale], G8 [Geriatric 8], GDS [Geriatric Depression Scale], MMSE [Mini Mental Status Examination], MNA [Mini-Nutritional Assessment], TGUG [Timed Get Up and Go], QLQ-C30 [EORTC Quality of Life Questionnaire-C30]; blue graph); FLOT = 5-fluorouracil, folinic acid, oxaliplatin and docetaxel, FOLFOX = folinic acid, 5-fluorouracil and oxaliplatin, FP = 5-FU/cisplatin, No neo adj. th. = no neoadjuvant therapy, RCTx = radiochemotherapy, significant differences between recommendations marked with an asterisk, n=17

Table S2: Comorbidities, prescriptions drugs and examinations of vignette A

**Comorbidities**	Two vessel coronary artery disease, mitral valve insufficiency II°, NYHA II, Diabetes mellitus type II, arterial hypertension, fatty liver disease I-II°, hyperthyroidism, hyperlipidemia, status post<br>status post posterior wall infarction, status post cataract surgery
**Prescription drugs**	Carvedilol 12.5mg, Losartan 5mg, Lercanidipine 20mg, Aspirin 100mg, Simvastatin 40mg, Pantoprazole 40mg, Thiamazole 10mg, Metamizole as needed
**Examinations**	CT: Tumor in the gastroesophageal junction with lumen-constricting wall thickening. No evidence of the presence of metastases

Table S3: Lab results of vignette A

**Laboratory test**	**Result**	**Reference range**
Hemoglobin	8.4 mmol/L	7.4-10.7 mmol/L
Leucocytes	8.49 GPt/L	3.8-9.8 GPt/L
Thrombocytes	265 GPt/L	150-400 GPt/L
Creatinine	75 µmol/L	44-80 µmol/L
eGFR	87 mL/min/1.73	
Urea	8.4 mmol/L	3.5-7.2 mmol/L
ALAT	0.45 µmol/L	< 0.85 µmol/L
ASAT	0.37 µmol/L	< 0.85 µmol/L
Gamma-GT	1.95 µmol/L	< 1.19 µmol/L
LDH	3.39 µmol/L	2.3-3.37 µmol/L
Bilirubin (total)	5.0 µmol/L	< 21 µmol/L

Table S4: Results of geriatric assessment of vignette A

Geriatric tool with reference range	Result
Barthel Index (BI): activities of daily living<br>100 points: completely independent, **85-95: selectively in need of help**, 35-80: in need of assistance, <30: care dependency	95/100 ■■■■■■■■■■
Cumulative Illness Rating Scale (CIRS): Comorbidities<br>0 = no comorbidities, 56 maximum points possible	17/56 ■■■■■■■□□□
G8: Geriatric screening tool for vulnerability<br>**≤14 = abnormal screening**	10/17 ■■■■■■■□□□
Geriatric Depression Scale (GDS)<br>**0-5: normal**, 6-10: mild depression, 11-15: severe depression	0/15 ■■■■■■■■■■
Mini-Mental-Status-Examination (MMSE)<br>30-24: no / subtle cognitive deficits, **23-18: mild cognitive deficits**, ≤17 severe cognitive deficits	23/30 ■■■■■■■■□□
Mini Nutritional Assessment (MNA)<br>24-30: normal nutritional status, **17-23.5: risk for malnutrition,** <17: malnutrition	20/30 ■■■■■□□□□□
Timed up and go test: Mobility test: get up from chair, walk 3m, return and sitz down, in seconds<br>**<10 seconds: no impairment**, 10-19 seconds: less mobile, 20-29 seconds: reduced mobility, >30 seconds: severely reduced mobility	9 ■■■■■■■■■■
Stair climb test (SCT) over 2 stories (7.7m) in seconds<br>(healthy individual: <30 seconds)	37 ■■■■■■■■□□
QLQ-C30: Summary Score<br>A high result means a **high level of functioning** and should be **regarded positively**	77/100 ■■■■■■■■□□
QLQ-C30: Functions<br>A high result means a **high level of functioning** and should be **regarded positively**	
Physical functioning	87/100 ■■■■■■■■■□
Role functioning	83/100 ■■■■■■■■□□
Emotional functioning	92/100 ■■■■■■■■■□
Cognitive functioning	83/100 ■■■■■■■■□□
Social functioning	67/100 ■■■■■■■□□□
QLQ-C30: Symptoms<br>A high result means a **high level of symptoms** and should be **regarded negatively**	
Fatigue	44/100 ■■■■□□□□□□
Nausea and vomiting	50/100 ■■■■■□□□□□
Pain	50/100 ■■■■■□□□□□
Dyspnea	0/100 □□□□□□□□□□
Insomnia	0/100 □□□□□□□□□□
Loss of appetite	67/100 ■■■■■■■□□□
Constipation	0/100 □□□□□□□□□□
Diarrhea	0/100 □□□□□□□□□□
Financial difficulties	0/100 □□□□□□□□□□

Vignette B

77 years old male patient with a carcinoma of the gastroesophageal junction AEG II (cT2 cNX cM0, glandular mucinous adenocarcinoma, HER2/neu: 1+).

Question: Neoadjuvant chemotherapy?

The following options were given:

1. No neoadjuvant therapy (no neo. adj. th.)
2. Folinic acid, 5-fluorouracil and oxaliplatin (FOLFOX)
3. 5-FU/cisplatin (FP) / capecitabine/cisplatin (XP)
4. 5-fluorouracil, folinic acid, oxaliplatin and docetaxel (FLOT)
5. Radiochemotherapy carboplatin / paclitaxel (RCTx)

Table S7 shows the descriptive statistics on the therapeutic recommendations followed by a graphical presentation. In the first graph, step 1 (50-years-old, no comorbidities, cross-sectional imaging, stage of disease) and step 2 (actual age, video, comorbidities, medication, lab results) are shown. In the second graph, step 2 and step 3 (results of GA) are shown.

Table S5: Recommended therapeutic regimes for vignette B

	**no neo. adj. th.**	**FOLFOX**	**FP/XP**	**FLOT**	**RCTx**
Guideline / tumor findings					
Mean ± SD	21 ± 36	17 ± 23	10 ± 20	77 ± 34	23 ± 32
Variance	1319	548	384	1169	1041
„Consultation / video“					
Mean ± SD	35 ± 35	42 ± 35	13 ± 21	38 ± 38	20 ± 30
Variance	1252	1241	434	1408	900
„Geriatric assessment“					
Mean ± SD	26 ± 37	37 ± 30	16 ± 23	52 ± 36	27 ± 34
Variance	1403	921	538	1295	1132

“Guideline / tumor findings“: 50-years-old patient without comorbidities, cross-sectional imaging, stage of disease provided; “consultation / video”: actual age, video, comorbidities, medication, lab results provided; “geriatric assessment”: BI [Barthel Index], CIRS [Cumulative Illness Rating Scale], G8 [Geriatric 8], GDS [Geriatric Depression Scale], MMSE [Mini Mental Status Examination], MNA [Mini-Nutritional Assessment], TGUG [Timed Get Up and Go], QLQ-C30 [EORTC Quality of Life Questionnaire-C30]). FLOT = 5-fluorouracil, folinic acid, oxaliplatin and docetaxel, FOLFOX = folinic acid, FP = 5-FU/cisplatin, 5-fluorouracil and oxaliplatin, no neo. adj. th. = no neoadjuvant therapy, RCTx = radiochemotherapy carboplatin / paclitaxel, XP = capecitabine/cisplatin, Her2+: Trastuzumab not standard of care, n= 20.

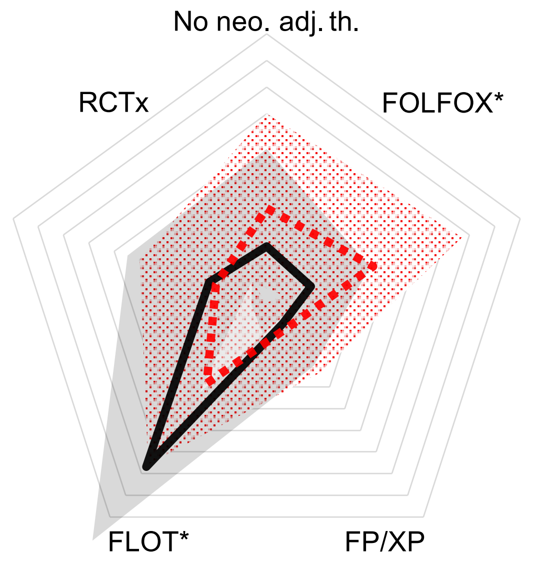

Figure 2: Radar plot for therapeutic recommendations for vignette B. The black and red lines show the median, the grey and light red areas the standard deviations of the treatment recommendations according to „guideline / tumor findings“ (“please assume 50-years-old patient without comorbidities”, cross-sectional imaging, stage of disease provided; black graph) and according to the “consultation / video” (actual age, video, comorbidities, medication, lab results provided; red graph); FLOT = 5-fluorouracil, folinic acid, oxaliplatin and docetaxel, FOLFOX = folinic acid, FP = 5-FU/cisplatin, 5-fluorouracil and oxaliplatin, no neo. adj. th. = no neoadjuvant therapy, RCTx = radiochemotherapy carboplatin / paclitaxel, XP = capecitabine/cisplatin, significant differences between recommendations marked with an asterisk, n= 20.

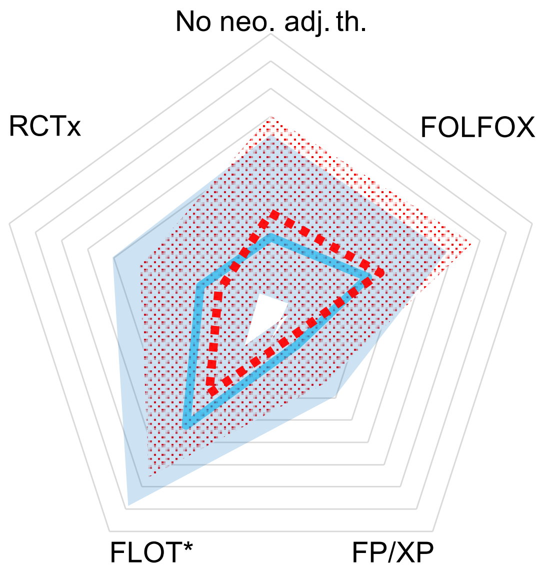

Figure 3: *Radar plot for therapeutic recommendations for vignette B. The red and blue lines show the median, the light red and light blue areas the standard deviations of the treatment recommendations according “consultation / video” (actual age, video, comorbidities, medication, lab results provided; red graph) and according to the additional results of the “geriatric assessment” (including BI [Barthel Index], CIRS [Cumulative Illness Rating Scale], G8 [Geriatric 8], GDS [Geriatric Depression Scale], MMSE [Mini Mental Status Examination], MNA [Mini-Nutritional Assessment], TGUG [Timed Get Up and Go], QLQ-C30 [EORTC Quality of Life Questionnaire-C30]; blue graph); FLOT = 5-fluorouracil, folinic acid, oxaliplatin and docetaxel, FOLFOX = folinic acid, FP = 5-FU/cisplatin, 5-fluorouracil and oxaliplatin, no neo. adj. th. = no neoadjuvant therapy, RCTx = radiochemotherapy carboplatin / paclitaxel, XP = capecitabine/cisplatin,* significant differences between recommendations marked with an asterisk, Her2+: Trastuzumab not standard of care, *n=20.*

Table S6: Comorbidities, prescriptions drugs and examinations of vignette

**Comorbidities**	Atrial fibrillation, status post ablation, intermittent atrioventricular block, pacemaker implantation, mechanical aortic valve replacement for 3rd degree aortic regurgitation, essential hypertension, hypothyroidism, diverticulosis, status post polypectomy in coecum and colon, status post cholecystectomy, status post hernioplasty for inguinal hernia
**Prescription drugs**	Phenprocoumon, Metoprolol 47.5 mg, Amlodipine 5mg, Valsartan 160mg, hydrochlorothiazide 12.5mg, Simvastatin 20mg, Levothyroxine 50µg, Esomeprazole 40 mg
**Examinations**	CT: No distant metastasis
	EGD: Tumor 42 to 39cm from alignment
	PET/CT: Malignant tumor in distal third of the esophagus. Pulmonary mass in Segment 6. Metastasis cannot be excluded.

Table S7: Lab results of vignette B

**Laboratory test**	**Result**	**Reference range**
Hemoglobin	8.3 mmol/L	7.4-10.7 mmol/L
Leucocytes	7.41 GPt/L	3.8-9.8 GPt/L
Thrombocytes	203 GPt/L	150-400 GPt/L
Creatinine	87 µmol/L	44-80 µmol/L
eGFR	74 mL/min/1.73	
Urea	5.7 mmol/L	3.5-7.2 mmol/L
ALAT	0.44 µmol/L	< 0.85 µmol/L
ASAT	0.38 µmol/L	< 0.85 µmol/L
Gamma-GT	0.67 µmol/L	< 1.19 µmol/L
LDH	4.62 µmol/L	2.3-3.37 µmol/L
Bilirubin (total)	6.6 µmol/L	< 21 µmol/L

Table S8: Results of geriatric assessment of vignette B

**Geriatric tool with reference range**	**Result**
Barthel Index (BI): activities of daily living<br>**100 points: completely independent**, 85-95: selectively in need of help, 35-80: in need of assistance, <30: care dependency	100/100 ■■■■■■■■■■
Cumulative Illness Rating Scale (CIRS): Comorbidities<br>0 = no comorbidities, 56 maximum points possible	16/56 ■■■■■■■□□□
G8: Geriatric screening tool for vulnerability<br>**≤14 = abnormal screening**	9/17 ■■■■■■□□□□
Geriatric Depression Scale (GDS)<br>**0-5: normal**, 6-10: mild depression, 11-15: severe depression	0/15 ■■■■■■■■■■
Mini-Mental-Status-Examination (MMSE)<br>**30-24: no / subtle cognitive deficits**, 23-18: mild cognitive deficits, ≤17 severe cognitive deficits	26/30 ■■■■■■■■■□
Mini Nutritional Assessment (MNA)<br>24-30: normal nutritional status, **17-23.5: risk for malnutrition**, <17: malnutrition	21/30 ■■■■■■□□□□
Timed up and go test: Mobility test: get up from chair, walk 3m, return and sitz down, in seconds<br>**<10 seconds: no impairment**, 10-19 seconds: less mobile, 20-29 seconds: reduced mobility, >30 seconds: severely reduced mobility	8 ■■■■■■■■■■
Stair climb test (SCT) over 2 stories (7.7m) in seconds (healthy individual: <30 seconds)	35 ■■■■■■■■■□
QLQ-C30: Summary Score<br>A high result means a **high level of functioning** and should be **regarded positively**	84/100 ■■■■■■■■□□
QLQ-C30: Functions<br>A high result means a **high level of functioning** and should be **regarded positively**	
Physical functioning	100/100 ■■■■■■■■■■
Role functioning	100/100 ■■■■■■■■■■
Emotional functioning	75/100 ■■■■■■■■□□
Cognitive functioning	100/100 ■■■■■■■■■■
Social functioning	100/100 ■■■■■■■■■■
QLQ-C30: Symptoms<br>A high result means a **high level of symptoms** and should be **regarded negatively**	
Fatigue	0/100 □□□□□□□□□□
Nausea and vomiting	0/100 □□□□□□□□□□
Pain	83/100 ■■■■■■■■□□
Dyspnea	33/100 ■■■□□□□□□□
Insomnia	67/100 ■■■■■■■□□□
Loss of appetite	0/100 □□□□□□□□□□
Constipation	0/100 □□□□□□□□□□
Diarrhea	0/100 □□□□□□□□□□
Financial difficulties	0/100 □□□□□□□□□□

Vignette C

76 years old male patient with a carcinoma of the gastroesophageal junction AEG II (cT3 N0 M0, adenocarcinoma, intestinal type (Laurèn classification), HER2/neu 3+, MSS, G2).

Question: Neoadjuvant chemotherapy?

The following options were given:

1. No neoadjuvant therapy (no neo. adj. th.)
2. Oxaliplatin/5-FU (Ox/FU)
3. 5-FU/cisplatin (FP) / capecitabine/cisplatin (XP)
4. 5-fluorouracil, folinic acid, oxaliplatin and docetaxel (FLOT)
5. Radiochemotherapy carboplatin / paclitaxel (RCTx)

Table S11 shows the descriptive statistics on the therapeutic recommendations followed by a graphical presentation. In the first graph, step 1 (50-years-old, no comorbidities, cross-sectional imaging, stage of disease) and step 2 (actual age, video, comorbidities, medication, lab results) are shown. In the second graph, step 2 and step 3 (results of GA) are shown.

Table S9: Recommended therapeutic regimes for vignette C

	**No neo. adj. th.**	**Ox/FU**	**FP/XP**	**FLOT**	**RCTx**
Guideline / tumor findings					
Mean ± SD	3 ± 7	6 ± 14	4 ± 8	97 ± 6	21 ± 27
Variance	46	202	61	42	721
„Consultation / video“					
Mean ± SD	12 ± 23	55 ± 39	3 ± 5	39 ± 38	24 ± 30
Variance	550	1498	23	1442	922
„Geriatric assessment“					
Mean ± SD	17 ± 27	58 ± 35	5 ± 9	35 ± 38	20 ± 29
Variance	745	1258	90	1412	866

“Guideline / tumor findings“: 50-years-old patient without comorbidities, cross-sectional imaging, stage of disease provided; “consultation / video”: actual age, video, comorbidities, medication, lab results provided; “geriatric assessment”: BI [Barthel Index], CIRS [Cumulative Illness Rating Scale], G8 [Geriatric 8], GDS [Geriatric Depression Scale], MMSE [Mini Mental Status Examination], MNA [Mini-Nutritional Assessment], TGUG [Timed Get Up and Go], QLQ-C30 [EORTC Quality of Life Questionnaire-C30]). FLOT = 5-fluorouracil, folinic acid, oxaliplatin and docetaxel, FP = 5-FU/cisplatin, no neo. adj. th. = no neoadjuvant therapy, Ox/FU = oxaliplatin/5-FU, RCTx = radiochemotherapy carboplatin / paclitaxel, XP = capecitabine/cisplatin, Her2+: Trastuzumab not standard of care, n= 15

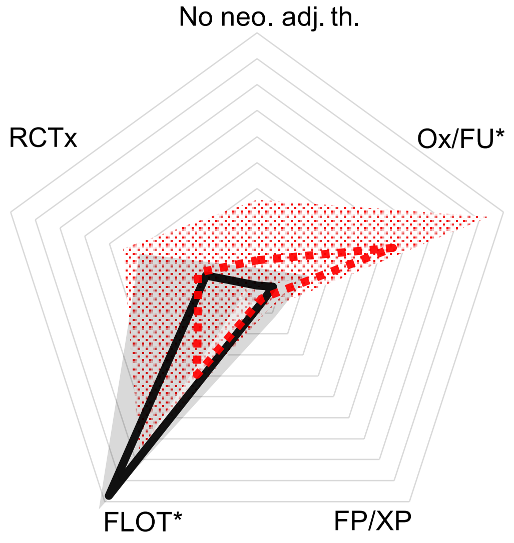

Figure 4: Radar plot for therapeutic recommendations for vignette C. The black and red lines show the median, the grey and light red areas the standard deviations of the treatment recommendations according to „guideline / tumor findings“ (“please assume 50-years-old patient without comorbidities”, cross-sectional imaging, stage of disease provided; black graph) and according to the “consultation / video” (actual age, video, comorbidities, medication, lab results provided; red graph); FLOT = 5-fluorouracil, folinic acid, oxaliplatin and docetaxel, FP = 5-FU/cisplatin, no neo. adj. th. = no neoadjuvant therapy, Ox/FU = oxaliplatin/5-FU, RCTx = radiochemotherapy carboplatin / paclitaxel, XP = capecitabine/cisplatin, significant differences between recommendations marked with an asterisk, Her2+: Trastuzumab not standard of care, n= 15

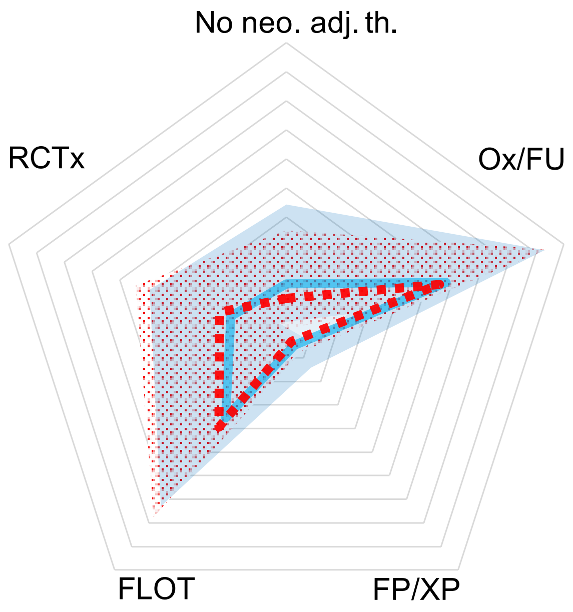

Figure 5: Radar plot for therapeutic recommendations for vignette C. The red and blue lines show the median, the light red and light blue areas the standard deviations of the treatment recommendations according “consultation / video” (actual age, video, comorbidities, medication, lab results provided; red graph) and according to the additional results of the “geriatric assessment” (including BI [Barthel Index], CIRS [Cumulative Illness Rating Scale], G8 [Geriatric 8], GDS [Geriatric Depression Scale], MMSE [Mini Mental Status Examination], MNA [Mini-Nutritional Assessment], TGUG [Timed Get Up and Go], QLQ-C30 [EORTC Quality of Life Questionnaire-C30]; blue graph); FLOT = 5-fluorouracil, folinic acid, oxaliplatin and docetaxel, FP = 5-FU/cisplatin, no neo. adj. th. = no neoadjuvant therapy, Ox/FU = oxaliplatin/5-FU, RCTx = radiochemotherapy carboplatin / paclitaxel, XP = capecitabine/cisplatin, significant differences between recommendations marked with an asterisk, Her2+: Trastuzumab not standard of care, n= 15

Table S10: Comorbidities, prescriptions drugs and examinations of vignette C

**Comorbidities**	Essential hypertension, hyperlipidemia, reflux esophagitis III°, benign prostatic hyperplasia, mild cognitive impairment after traffic accident with traumatic brain injury 30 years ago, status post tympanic membrane surgery 25 years ago.
**Prescription drugs**	Amlodipine 10mg, Losartan 100mg, Bisoprolol 10mg, hydrochlorothiazide 12.5mg, Esomeprazole 40mg, Tapentadol as needed
**Examinations**	CT: Malignant tumor of the aboral esophagus involving the cardia of the stomach. No definite evidence of metastatic lymph nodes. No evidence of distant metastases.
	Esophagogastroduodenoscopy: Elongated tumor in the cardia.

Table S11: Lab results of vignette C

**Laboratory test**	**Result**	**Reference range**
Hemoglobin	10.1 mmol/L	7.4-10.7 mmol/L
Leucocytes	9.14 GPt/L	3.8-9.8 GPt/L
Thrombocytes	383 GPt/L	150-400 GPt/L
Creatinine	104 µmol/L	44-80 µmol/L
eGFR	71 mL/min/1.73	
Urea	7.0 mmol/L	3.5-7.2 mmol/L
ALAT	0.32 µmol/L	< 0.85 µmol/L
ASAT	0.33 µmol/L	< 0.85 µmol/L
Gamma-GT	0.56 µmol/L	< 1.19 µmol/L
LDH	3.5 µmol/L	2.3-3.37 µmol/L
Bilirubin (total)	16.8 µmol/L	< 21 µmol/L

Table S12: Results of geriatric assessment of vignette C

Geriatric tool with reference range	Result
Barthel Index (BI): activities of daily living<br>**100 points: completely independent**, 85-95: selectively in need of help, 35-80: in need of assistance, <30: care dependency	100/100 ■■■■■■■■■■
Cumulative Illness Rating Scale (CIRS): Comorbidities<br>0 = no comorbidities, 56 maximum points possible	13/56 ■■■■■■■■□□
G8: Geriatric screening tool for vulnerability<br>**≤14 = abnormal screening**	7.5/17 ■■■■■□□□□□
Geriatric Depression Scale (GDS)<br>**0-5: normal**, 6-10: mild depression, 11-15: severe depression	2/15 ■■■■■■■■■■
Mini-Mental-Status-Examination (MMSE)<br>**30-24: no / subtle cognitive deficits**, 23-18: mild cognitive deficits, ≤17 severe cognitive deficits	28/30 ■■■■■■■■■■
Mini Nutritional Assessment (MNA)<br>24-30: normal nutritional status, **17-23.5: risk for malnutrition**, <17: malnutrition	19/30 ■■■□□□□□□□
Timed up and go test: Mobility test: get up from chair, walk 3m, return and sitz down, in seconds<br>**<10 seconds: no impairment**, 10-19 seconds: less mobile, 20-29 seconds: reduced mobility, >30 seconds: severely reduced mobility	9 ■■■■■■■■■■
Stair climb test (SCT) over 2 stories (7.7m) in seconds<br>(healthy individual: <30 seconds)	38 ■■■■■■■■□□
QLQ-C30: Summary Score<br>A high result means a **high level of functioning** and should be **regarded positively**	79/100 ■■■■■■■■□□
QLQ-C30: Functions<br>A high result means a **high level of functioning** and should be **regarded positively**	
Physical functioning	73/100 ■■■■■■■□□□
Role functioning	83/100 ■■■■■■■■□□
Emotional functioning	92/100 ■■■■■■■■■□
Cognitive functioning	67/100 ■■■■■■■□□□
Social functioning	67/100 ■■■■■■■□□□
QLQ-C30: Symptoms<br>A high result means a **high level of symptoms** and should be **regarded negatively**	
Fatigue	44/100 ■■■■□□□□□□
Nausea and vomiting	0/100 □□□□□□□□□□
Pain	50/100 ■■■■■□□□□□
Dyspnea	0/100 □□□□□□□□□□
Insomnia	0/100 □□□□□□□□□□
Loss of appetite	67/100 ■■■■■■■□□□
Constipation	0/100 □□□□□□□□□□
Diarrhea	0/100 □□□□□□□□□□
Financial difficulties	0/100 □□□□□□□□□□

Vignette D

75 years old female patient with an adenocarcinoma of the gastroesophageal junction AEG II (cT4a cN0 cM0, moderately differentiated, mucinous adenocarcinoma, intestinal type, Her2/neu-negative, G2).

Question: Neoadjuvant chemotherapy?

The following options were given:

- No neo adjuvant therapy (no neo adj. th.)
- Folinic acid, 5-fluorouracil and oxaliplatin (FOLFOX)
- 5-FU/cisplatin (FP)
- 5-fluorouracil, folinic acid, oxaliplatin and docetaxel (FLOT)
- Radiochemotherapy (RCTx)

Table S15 shows the descriptive statistics on the therapeutic recommendations followed by a graphical presentation. In the first graph, step 1 (50-years-old, no comorbidities, cross-sectional imaging, stage of disease) and step 2 (actual age, video, comorbidities, medication, lab results) are shown. In the second graph, step 2 and step 3 (results of GA) are shown.

Table S13: Recommended therapeutic regimes for vignette D

	**no neo adj. th.**	**FOLFOX**	**FP**	**FLOT**	**RCTx**
Guideline / tumor findings					
Mean ± SD	1 ± 1	20 ± 29	8 ± 19	94 ± 16	11 ± 20
Variance	1	849	350	247	418
„Consultation / video“					
Mean ± SD	18 ± 28	33 ± 37	4 ± 12	40 ± 38	28 ± 35
Variance	785	1374	143	1434	1225
„Geriatric assessment“					
Mean ± SD	21 ± 30	34 ± 42	3 ± 8	50 ± 38	21 ± 30
Variance	877	1801	59	1471	880

“Guideline / tumor findings“: 50-years-old patient without comorbidities, cross-sectional imaging, stage of disease provided; “consultation / video”: actual age, video, comorbidities, medication, lab results provided; “geriatric assessment”: BI [Barthel Index], CIRS [Cumulative Illness Rating Scale], G8 [Geriatric 8], GDS [Geriatric Depression Scale], MMSE [Mini Mental Status Examination], MNA [Mini-Nutritional Assessment], TGUG [Timed Get Up and Go], QLQ-C30 [EORTC Quality of Life Questionnaire-C30]). FLOT= 5-fluorouracil, folinic acid, oxaliplatin and docetaxel, FOLFOX = folinic acid, 5-fluorouracil and oxaliplatin, FP = 5-FU/cisplatin, No neo adj. th.= no neo adjuvant therapy, RCTx = radiochemotherapy, n= 9

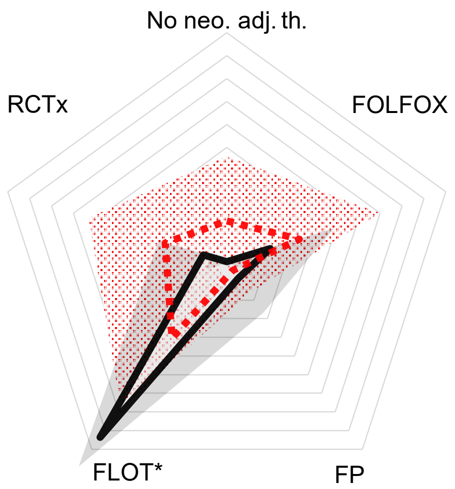

Figure 6: Radar plot for therapeutic recommendations for vignette D. The black and red lines show the median, the grey and light red areas the standard deviations of the treatment recommendations according to „guideline / tumor findings" ("please assume 50-years-old patient without comorbidities", cross-sectional imaging, stage of disease provided; black graph) and according to the "consultation / video" (actual age, video, comorbidities, medication, lab results provided; red graph); no neo adj. th.= no neo adjuvant therapy, FLOT= 5-fluorouracil, folinic acid, oxaliplatin and docetaxel, FOLFOX = folinic acid, 5-fluorouracil and oxaliplatin, FP = 5-FU/cisplatin, No neo adj. th.= no neo adjuvant therapy, RCTx = radiochemotherapy, significant differences between recommendations marked with an asterisk, n= 9

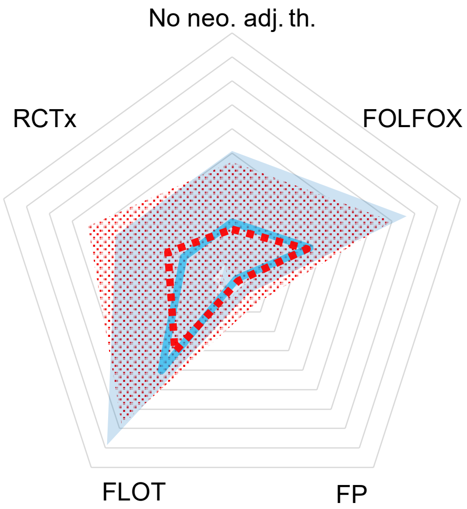

Figure 7: Radar plot for therapeutic recommendations for vignette D. The red and blue lines show the median, the light red and light blue areas the standard deviations of the treatment recommendations according "consultation / video" (actual age, video, comorbidities, medication, lab results provided; red graph) and according to the additional results of the "geriatric assessment" (including BI [Barthel Index], CIRS [Cumulative Illness Rating Scale], G8 [Geriatric 8], GDS [Geriatric Depression Scale], MMSE [Mini Mental Status Examination], MNA [Mini-Nutritional Assessment], TGUG [Timed Get Up and Go], QLQ-C30 [EORTC Quality of Life Questionnaire-C30]; blue graph); FLOT= 5-fluorouracil, folinic acid, oxaliplatin and docetaxel, FOLFOX = folinic acid, 5-fluorouracil and oxaliplatin, FP = 5-FU/cisplatin, No neo adj. th.= no neo adjuvant therapy, RCTx = radiochemotherapy, significant differences between recommendations marked with an asterisk, n= 9

Table S14: Comorbidities, prescriptions drugs and examinations of vignette D

**Comorbidities**	Essential hypertension, type 2 diabetes, morbid obesity: BMI 38, hyperthyroidism, hyperuricemia, chronic low back pain, uterine fibroid, status post total hip replacement, status post cholecystectomy
**Prescription drugs**	Lercanidipine 10mg, Losartan 100mg, hydrochlorothiazide 12.5mg, L-Thyroxin 75µg/ potassium iodide 196µg, Allopurinol 300mg, Sitagliptin 128mg, Tilidin/Naloxon 50/4mg, Pantoprazole 20mg
**Examinations**	CT: No distant metastases.
	Locally advanced, hypermetabolic tumor at the gastroesophageal junction. Suspect as a mediastinal lymph node metastasis, differential diagnosis: diagnosed thymic cyst.
	EGD: Ulcerated carcinoma with infiltrative growth, approximately 35 cm to 43 cm from alignment.
	Laparotomy: No peritoneal carcinomatosis

Table S15: Lab results of vignette D

**Laboratory test**	**Result**	**Reference range**
Hemoglobin	7.1 mmol/L	7.4-10.7 mmol/L
Leucocytes	5.75 GPt/L	3.8-9.8 GPt/L
Thrombocytes	317 GPt/L	150-400 GPt/L
Creatinine	107 µmol/L	44-80 µmol/L
eGFR	44 mL/min/1.73	
Urea	8.1 mmol/L	3.5-7.2 mmol/L
ALAT	0.15 µmol/L	< 0.85 µmol/L
ASAT	0.34 µmol/L	< 0.85 µmol/L
Gamma-GT	0.23 µmol/L	< 1.19 µmol/L
LDH	3.93 µmol/L	2.3-3.37 µmol/L
Bilirubin (total)	7.2 µmol/L	< 21 µmol/L

Table S16: Results of geriatric assessment of vignette D

Geriatric tool with reference range	Result
Barthel Index (BI): activities of daily living<br>**100 points: completely independent**, 85-95: selectively in need of help, 35-80: in need of assistance, <30: care dependency	100/100 ■■■■■■■■■■
Cumulative Illness Rating Scale (CIRS): Comorbidities<br>0 = no comorbidities, 56 maximum points possible	14/56 ■■■■■■■■□□
G8: Geriatric screening tool for vulnerability<br>**≤14 = abnormal screening**	11/17 ■■■■■■■■□□
Geriatric Depression Scale (GDS)<br>**0-5: normal**, 6-10: mild depression, 11-15: severe depression	1/15 ■■■■■■■■■■
Mini-Mental-Status-Examination (MMSE)<br>**30-24: no / subtle cognitive deficits**, 23-18: mild cognitive deficits, ≤17 severe cognitive deficits	27/30 ■■■■■■■■■■
Mini Nutritional Assessment (MNA)<br>24-30: normal nutritional status, **17-23.5: risk for malnutrition**, <17: malnutrition	20.5/30 ■■■■■□□□□□
Timed up and go test: Mobility test: get up from chair, walk 3m, return and sitz down, in seconds<br><10 seconds: no impairment, **10-19 seconds: less mobile**, 20-29 seconds: reduced mobility, >30 seconds: severely reduced mobility	12 ■■■■■■■■■□
Stair climb test (SCT) over 2 stories (7.7m) in seconds<br>(healthy individual: <30 seconds)	n.a.
QLQ-C30: Summary Score<br>A high result means a **high level of functioning** and should be **regarded positively**	86/100 ■■■■■■■■■□
QLQ-C30: Functions<br>A high result means a **high level of functioning** and should be **regarded positively**	
Physical functioning	87/100 ■■■■■■■■■□
Role functioning	100/100 ■■■■■■■■■■
Emotional functioning	58/100 ■■■■■■□□□□
Cognitive functioning	100/100 ■■■■■■■■■■
Social functioning	100/100 ■■■■■■■■■■
QLQ-C30: Symptoms<br>A high result means a **high level of symptoms** and should be **regarded negatively**	
Fatigue	33/100 ■■■□□□□□□□
Nausea and vomiting	0/100 □□□□□□□□□□
Pain	0/100 □□□□□□□□□□
Dyspnea	0/100 □□□□□□□□□□
Insomnia	0/100 □□□□□□□□□□
Loss of appetite	0/100 □□□□□□□□□□
Constipation	100/100 ■■■■■■■■■■
Diarrhea	0/100 □□□□□□□□□□
Financial difficulties	0/100 □□□□□□□□□□

Vignette E

79 years old male patient with a gastric carcinoma with liver metastasis (moderately differentiated tubular adenocarcinoma). First line therapy with oxaliplatin / 5-FU.

Question: Palliative 2nd line chemotherapy?

The following options were given:

1. Best supportive care only (BSC)
2. Ramucirumab monotherapy (Ramu)
3. Irinotecan (Iri)
4. Ramucirumab/paclitaxel (Ramu/Pac)

Table S19 shows the descriptive statistics on the therapeutic recommendations followed by a graphical presentation. In the first graph, step 1 (50-years-old, no comorbidities, cross-sectional imaging, stage of disease) and step 2 (actual age, video, comorbidities, medication, lab results) are shown. In the second graph, step 2 and step 3 (results of GA) are shown.

Table S17: Recommended therapeutic regimes for vignette E

	**BSC**	**Ramu**	**Iri**	**Ramu/Pac**
Guideline / tumor findings				
Mean ± SD	1 ± 2	9 ± 18	60 ± 28	79 ± 20
Variance	3	308	760	386
„Consultation / video"				
Mean ± SD	26 ± 33	40 ± 40	36 ± 29	46 ± 35
Variance	1115	1622	854	1256
„Geriatric assessment"				
Mean ± SD	24 ± 35	35 ± 36	38 ± 34	44 ± 32
Variance	1224	1309	1130	1000

"Guideline / tumor findings": 50-years-old patient without comorbidities, cross-sectional imaging, stage of disease provided; "consultation / video": actual age, video, comorbidities, medication, lab results provided; "geriatric assessment": BI [Barthel Index], CIRS [Cumulative Illness Rating Scale], G8 [Geriatric 8], GDS [Geriatric Depression Scale], MMSE [Mini Mental Status Examination], MNA [Mini-Nutritional Assessment], TGUG [Timed Get Up and Go], QLQ-C30 [EORTC Quality of Life Questionnaire-C30]). BSC = best supportive care only, Iri = irinotecan, Ramu = ramucirumab monotherapy, Ramu/Pac = ramucirumab/paclitaxel, n= 13

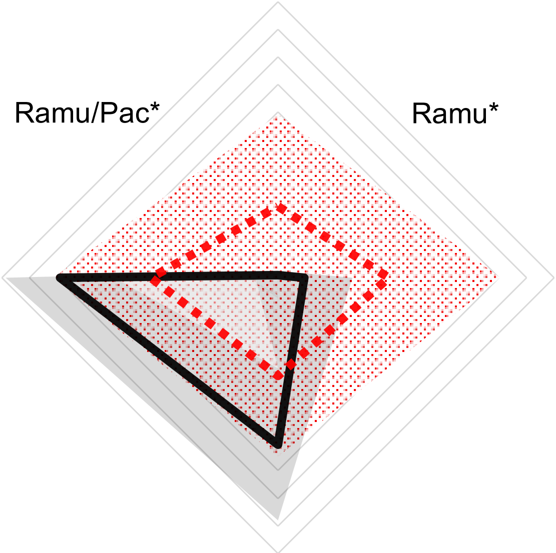

Figure 8: Radar plot for therapeutic recommendations for vignette E. The black and red lines show the median, the grey and light red areas the standard deviations of the treatment recommendations according to „guideline / tumor findings“ (“please assume 50-years-old patient without comorbidities”, cross-sectional imaging, stage of disease provided; black graph) and according to the “consultation / video” (actual age, video, comorbidities, medication, lab results provided; red graph); BSC = best supportive care only, Iri = irinotecan, Ramu = ramucirumab monotherapy, Ramu/Pac = ramucirumab/paclitaxel, significant differences between recommendations marked with an asterisk, n= 13

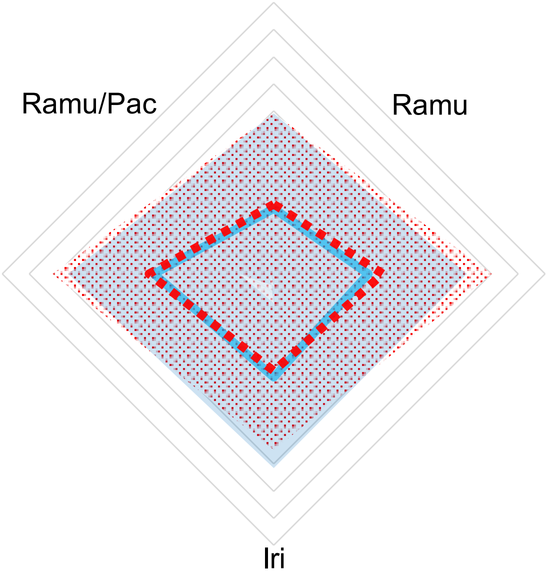

Figure 9: Radar plot for therapeutic recommendations for vignette E. The black and red lines show the median, the grey and light red areas the standard deviations of the treatment recommendations according to „guideline / tumor findings“ (“please assume 50-years-old patient without comorbidities”, cross-sectional imaging, stage of disease provided; black graph) and according to the “consultation / video” (actual age, video, comorbidities, medication, lab results provided; red graph); BSC = best supportive care only, Iri = irinotecan, Ramu = ramucirumab monotherapy, Ramu/Pac = ramucirumab/paclitaxel, significant differences between recommendations marked with an asterisk, n= 13

Table S18: Comorbidities, prescriptions drugs and examinations of vignette E

**Comorbidities**	Essential hypertension, moderate aortic stenosis, type-c gastritis, sigma diverticulitis, status post cholecystectomy, status post knee replacement
**Prescription drugs**	Aspirin 100mg, Creon 25.000, L-Thyroxin 50µg, Mirtazapine 15mg, Torsemide 10mg
**Examinations**	CT: Hepatic metastasis in liver segments 4, 7 and 8.

Table S19: Comorbidities, prescriptions drugs and examinations of vignette E

**Laboratory test**	**Result**	**Reference range**
Hemoglobin	7.8 mmol/L	7.4-10.7 mmol/L
Leucocytes	8.49 GPt/L	3.8-9.8 GPt/L
Thrombocytes	185 GPt/L	150-400 GPt/L
Creatinine	70 µmol/L	44-80 µmol/L
eGFR	86 mL/min/1.73	
Urea	5.7 mmol/L	3.5-7.2 mmol/L
ALAT	0.52 µmol/L	< 0.85 µmol/L
ASAT	0.48 µmol/L	< 0.85 µmol/L
Gamma-GT	0.6 µmol/L	< 1.19 µmol/L
LDH	6.54 µmol/L	2.3-3.37 µmol/L
Bilirubin (total)	6.2 µmol/L	< 21 µmol/L

Table S20: Results of geriatric assessment of vignette

**Geriatric tool with reference range**	**Result**
Barthel Index (BI): activities of daily living<br>**100 points: completely independent**, 85-95: selectively in need of help, 35-80: in need of assistance, <30: care dependency	95/100 ■■■■■■■■■■
Cumulative Illness Rating Scale (CIRS): Comorbidities<br>0 = no comorbidities, 56 maximum points possible	18/56 ■■■■■■■□□□
G8: Geriatric screening tool for vulnerability<br>**≤14 = abnormal screening**	9/17 ■■■■■■□□□□
Geriatric Depression Scale (GDS)<br>0-5: normal, **6-10: mild depression**, 11-15: severe depression	8/15 ■■■■■■■□□□
Mini-Mental-Status-Examination (MMSE)<br>**30-24: no / subtle cognitive deficits**, 23-18: mild cognitive deficits, ≤17 severe cognitive deficits	27/30 ■■■■■■■■■■
Mini Nutritional Assessment (MNA)<br>24-30: normal nutritional status, **17-23.5: risk for malnutrition**, <17: malnutrition	20/30 ■■■■■□□□□□
Timed up and go test: Mobility test: get up from chair, walk 3m, return and sitz down, in seconds<br><10 seconds: no impairment, **10-19 seconds: less mobile**, 20-29 seconds: reduced mobility, >30 seconds: severely reduced mobility	10 ■■■■■■■■■□
Stair climb test (SCT) over 2 stories (7.7m) in seconds<br>(healthy individual: <30 seconds)	44 ■■■■■■■□□□
QLQ-C30: Summary Score<br>A high result means a **high level of functioning** and should be **regarded positively**	65/100 ■■■■■■■□□□
QLQ-C30: Functions<br>A high result means a **high level of functioning** and should be **regarded positively**	
Physical functioning	100/100 ■■■■■■■■■■
Role functioning	33/100 ■■■□□□□□□□
Emotional functioning	50/100 ■■■■■□□□□□
Cognitive functioning	100/100 ■■■■■■■■■■
Social functioning	33/100 ■■■□□□□□□□
QLQ-C30: Symptoms<br>A high result means a **high level of symptoms** and should be **regarded negatively**	
Fatigue	44/100 ■■■■□□□□□□
Nausea and vomiting	33/100 ■■■□□□□□□□
Pain	67/100 ■■■■■■■□□□
Dyspnea	0/100 □□□□□□□□□□
Insomnia	0/100 □□□□□□□□□□
Loss of appetite	100/100 ■■■■■■■■■■
Constipation	0/100 □□□□□□□□□□
Diarrhea	33/100 ■■■□□□□□□□
Financial difficulties	0/100 □□□□□□□□□□

Vignette F

84 years old female patient with a locally advanced pancreatic head carcinoma (cT2 cN0 cMx, moderately differentiated ductal adenocarcinoma, G2).

Question: Neoadjuvant chemotherapy?

The following options were given:

1. Best supportive care only (BSC)
2. Gemcitabine (Gem)
3. Gemcitabine/nab-paclitaxel (Gem/nPac)
4. Folinic acid, 5-fluorouracil, oxaliplatin and irinotecan (FOLFOXIRI)
5. Folinic acid, 5-fluorouracil and oxaliplatin (FOLFOX)
6. Radiochemotherapy (RCTx)

Table S23 shows the descriptive statistics on the therapeutic recommendations followed by a graphical presentation. In the first graph, step 1 (50-years-old, no comorbidities, cross-sectional imaging, stage of disease) and step 2 (actual age, video, comorbidities, medication, lab results) are shown. In the second graph, step 2 and step 3 (results of GA) are shown.

Table S21: Recommended therapeutic regimes for vignette F

	**BSC**	**Gem**	**Gem/nPac**	**FOLFOXIRI**	**FOLFOX**	**RCTx**
Guideline / tumor findings						
Mean ± SD	5 ± 17	14 ± 31	25 ± 34	85 ± 27	14 ± 23	16 ± 28
Variance	297	960	1187	748	537	759
„Consultation / video“						
Mean ± SD	15 ± 23	49 ± 32	58 ± 37	15 ± 30	15 ± 25	4 ± 12
Variance	525	994	1350	927	650	146
„Geriatric assessment“						
Mean ± SD	23 ± 28	57 ± 32	48 ± 37	6 ± 14	12 ± 21	3 ± 12
Variance	808	1051	1360	193	448	138

“Guideline / tumor findings“: 50-years-old patient without comorbidities, cross-sectional imaging, stage of disease provided; “consultation / video”: actual age, video, comorbidities, medication, lab results provided; “geriatric assessment”: BI [Barthel Index], CIRS [Cumulative Illness Rating Scale], G8 [Geriatric 8], GDS [Geriatric Depression Scale], MMSE [Mini Mental Status Examination], MNA [Mini-Nutritional Assessment], TGUG [Timed Get Up and Go], QLQ-C30 [EORTC Quality of Life Questionnaire-C30]). BSC = best supportive care only, FOLFOX = folinic acid, 5-fluorouracil and oxaliplatin, FOLFOXIRI = folinic acid, 5-fluorouracil, oxaliplatin and irinotecan, Gem = gemcitabine, Gem/nPac = gemcitabine/nab-paclitaxel, RCTx = radiochemotherapy, n= 19

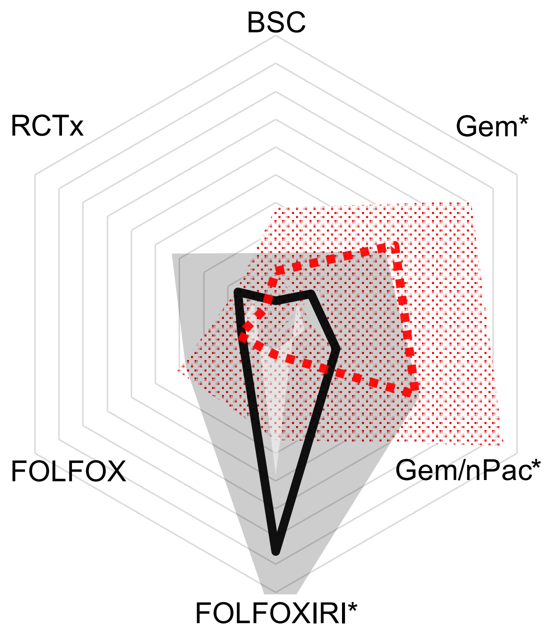

Figure 10: Radar plot for therapeutic recommendations for vignette F. The black and red lines show the median, the grey and light red areas the standard deviations of the treatment recommendations according to „guideline / tumor findings“ (“please assume 50-years-old patient without comorbidities”, cross-sectional imaging, stage of disease provided; black graph) and according to the “consultation / video” (actual age, video, comorbidities, medication, lab results provided; red graph); BSC = best supportive care only, FOLFOX = folinic acid, 5-fluorouracil and oxaliplatin, FOLFOXIRI = folinic acid, 5-fluorouracil, oxaliplatin and irinotecan, Gem = gemcitabine, Gem/nPac = gemcitabine/nab-paclitaxel, RCTx = radiochemotherapy, significant differences between recommendations marked with an asterisk, n= 19

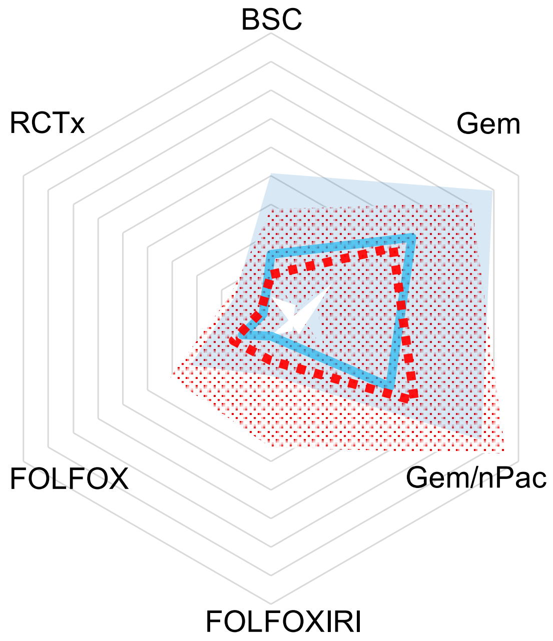

Figure 11: Radar plot for therapeutic recommendations for vignette F. The red and blue lines show the median, the light red and light blue areas the standard deviations of the treatment recommendations according “consultation / video” (actual age, video, comorbidities, medication, lab results provided; red graph) and according to the additional results of the “geriatric assessment” (including BI [Barthel Index], CIRS [Cumulative Illness Rating Scale], G8 [Geriatric 8], GDS [Geriatric Depression Scale], MMSE [Mini Mental Status Examination], MNA [Mini-Nutritional Assessment], TGUG [Timed Get Up and Go], QLQ-C30 [EORTC Quality of Life Questionnaire-C30]; blue graph); BSC = best supportive care only, FOLFOX = folinic acid, 5-fluorouracil and oxaliplatin, FOLFOXIRI = folinic acid, 5-fluorouracil, oxaliplatin and irinotecan, Gem = gemcitabine, Gem/nPac = gemcitabine/nab-paclitaxel, RCTx = radiochemotherapy, significant differences between recommendations marked with an asterisk, n= 19

Table S22: Comorbidities, prescriptions drugs and examinations of vignette F

**Comorbidities**	Essential hypertension, type 2 diabetes mellitus, hypercholesterolemia, diverticulosis, polyps in sigmoid colon (tubulovillous adenoma), Struma diffusa et nodosa, adrenal adenoma on the left
**Prescription drugs**	Metoprolol 47.5mg, Candesartan 16mg, Simvastatin 20mg, Pantoprazole 20mg, ASS 100, Insulin detemir, Insulin lispro, Metamizole as needed
**Examinations**	CT: Pancreatic cancer. Infiltration of the common hepatic artery and of the splenic artery. Adrenal adenoma on the left. Suspected renal cell carcinoma (not the oncological focus). Hemangioma in liver segment VII. No evidence of distant metastases.
	Abdominal ultrasound: Tumor of the pancreatic head/corpus: diameter 4.5cm. Tumor of the right kidney, diameter approx. 6cm.
	Gastroscopy: No infiltration or impression of a tumor.
	Colonoscopy: tubulovillous adenoma with low-grade IEN.

Table S23: Lab results of vignette F

Laboratory test	Result	Reference range
Hemoglobin	7.8 mmol/L	7.4-10.7 mmol/L
Leucocytes	8.3 GPt/L	3.8-9.8 GPt/L
Thrombocytes	268 GPt/L	150-400 GPt/L
Creatinine	72 µmol/L	44-80 µmol/L
eGFR	66 mL/min/1.73	
Urea	n.a.	3.5-7.2 mmol/L
ALAT	0.21 µmol/L	< 0.85 µmol/L
ASAT	0.31 µmol/L	< 0.85 µmol/L
Gamma-GT	0.66 µmol/L	< 1.19 µmol/L
LDH	7.07 µmol/L	2.3-3.37 µmol/L
Bilirubin (total)	5.5 µmol/L	< 21 µmol/L

Table S24: Results of geriatric assessment of vignette F

Geriatric tool with reference range	Result
Barthel Index (BI): activities of daily living<br>100 points: completely independent, **85-95: selectively in need of help**, 35-80: in need of assistance, <30: care dependency	90/100 ■■■■■■■■■□
Cumulative Illness Rating Scale (CIRS): Comorbidities<br>0 = no comorbidities, 56 maximum points possible	19/56 ■■■■■■■□□□
G8: Geriatric screening tool for vulnerability<br>**≤14 = abnormal screening**	6/17 ■■■■□□□□□□
Geriatric Depression Scale (GDS)<br>0-5: normal, **6-10: mild depression**, 11-15: severe depression	8/15 ■■■■■■■□□□
Mini-Mental-Status-Examination (MMSE)<br>**30-24: no / subtle cognitive deficits**, 23-18: mild cognitive deficits, ≤17 severe cognitive deficits	24/30 ■■■■■■■■□□
Mini Nutritional Assessment (MNA)<br>24-30: normal nutritional status, 17-23.5: risk for malnutrition, **<17: malnutrition**	15.5/30 ■□□□□□□□□□
Timed up and go test: Mobility test: get up from chair, walk 3m, return and sitz down, in seconds<br><10 seconds: no impairment, **10-19 seconds: less mobile**, 20-29 seconds: reduced mobility, >30 seconds: severely reduced mobility	13 ■■■■■■■■■□
Stair climb test (SCT) over 2 stories (7.7m) in seconds<br>(healthy individual: <30 seconds)	49 ■■■■■■□□□□
QLQ-C30: Summary Score<br>A high result means a **high level of functioning** and should be **regarded positively**	54/100 ■■■■■□□□□□
QLQ-C30: Functions<br>A high result means a **high level of functioning** and should be **regarded positively**	
Physical functioning	73/100 ■■■■■■■□□□
Role functioning	83/100 ■■■■■■■■□□
Emotional functioning	33/100 ■■■□□□□□□□
Cognitive functioning	67/100 ■■■■■■■□□□
Social functioning	33/100 ■■■□□□□□□□
QLQ-C30: Symptoms<br>A high result means a **high level of symptoms** and should be **regarded negatively**	
Fatigue	67/100 ■■■■■■■□□□
Nausea and vomiting	0/100 □□□□□□□□□□
Pain	83/100 ■■■■■■■■□□
Dyspnea	33/100 ■■■□□□□□□□
Insomnia	67/100 ■■■■■■■□□□
Loss of appetite	100/100 ■■■■■■■■■■
Constipation	33/100 ■■■□□□□□□□
Diarrhea	0/100 □□□□□□□□□□
Financial difficulties	0/100 □□□□□□□□□□

Vignette G

76 years old male patient with metastatic pancreatic cancer (cM1, moderately differentiated ductal adenocarcinoma, MSS).

Question: Palliative chemotherapy?

The following options were given:

1. Best supportive care only (BSC)
1. Gemcitabine (Gem)
2. Gemcitabine/nab-paclitaxel (Gem/nPac)
3. Folinic acid, 5-fluorouracil, oxaliplatin and irinotecan (FOLFOXIRI)
4. Folinic acid, 5-fluorouracil and oxaliplatin (FOLFOX)

Table S27 shows the descriptive statistics on the therapeutic recommendations followed by a graphical presentation. In the first graph, step 1 (50-years-old, no comorbidities, cross-sectional imaging, stage of disease) and step 2 (actual age, video, comorbidities, medication, lab results) are shown. In the second graph, step 2 and step 3 (results of GA) are shown.

Table S25: Recommended therapeutic regimes for vignette G

	**BSC**	**Gem**	**Gem/nPac**	**FOLFOXIRI**	**FOLFOX**
Guideline /					
Mean ± SD	3 ± 6	7 ± 10	55 ± 35	97 ± 6	12 ± 21
Variance	32	92	1238	33	435
„Consultation / video“					
Mean ± SD	13 ± 17	42 ± 35	67 ± 30	17 ± 25	17 ± 26
Variance	293	1224	927	643	690
„Geriatric					
Mean ± SD	10 ± 18	41 ± 38	71 ± 32	19 ± 28	15 ± 26
Variance	329	1477	1040	782	674

“Guideline / tumor findings“: 50-years-old patient without comorbidities, cross-sectional imaging, stage of disease provided; “consultation / video”: actual age, video, comorbidities, medication, lab results provided; “geriatric assessment”: BI [Barthel Index], CIRS [Cumulative Illness Rating Scale], G8 [Geriatric 8], GDS [Geriatric Depression Scale], MMSE [Mini Mental Status Examination], MNA [Mini-Nutritional Assessment], TGUG [Timed Get Up and Go], QLQ-C30 [EORTC Quality of Life Questionnaire-C30]). BSC = best supportive care only, FOLFOX = folinic acid, 5-fluorouracil and oxaliplatin, FOLFOXIRI = folinic acid, 5-fluorouracil, oxaliplatin and irinotecan, Gem = gemcitabine, Gem/nPac = gemcitabine/nab-paclitaxel, n= 15

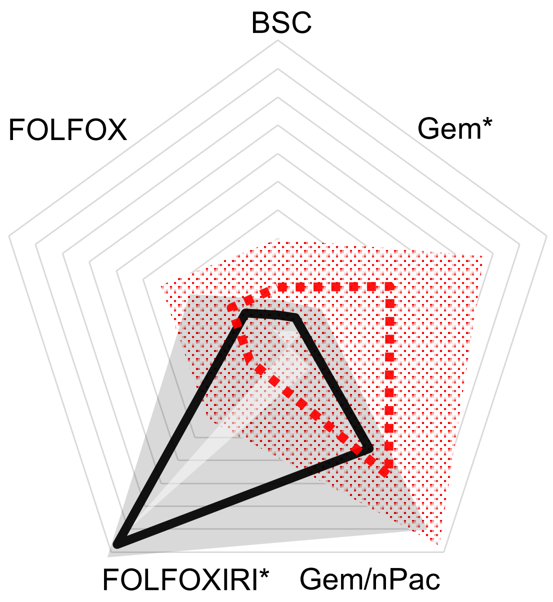

Figure 12: Radar plot for therapeutic recommendations for vignette G. The black and red lines show the median, the grey and light red areas the standard deviations of the treatment recommendations according to „guideline / tumor findings“ (“please assume 50-years-old patient without comorbidities”, cross-sectional imaging, stage of disease provided; black graph) and according to the “consultation / video” (actual age, video, comorbidities, medication, lab results provided; red graph); BSC = best supportive care only, FOLFOX = folinic acid, 5-fluorouracil and oxaliplatin, FOLFOXIRI = folinic acid, 5-fluorouracil, oxaliplatin and irinotecan, Gem = gemcitabine, Gem/nPac = gemcitabine/nab-paclitaxel, significant differences between recommendations marked with an asterisk, n= 15

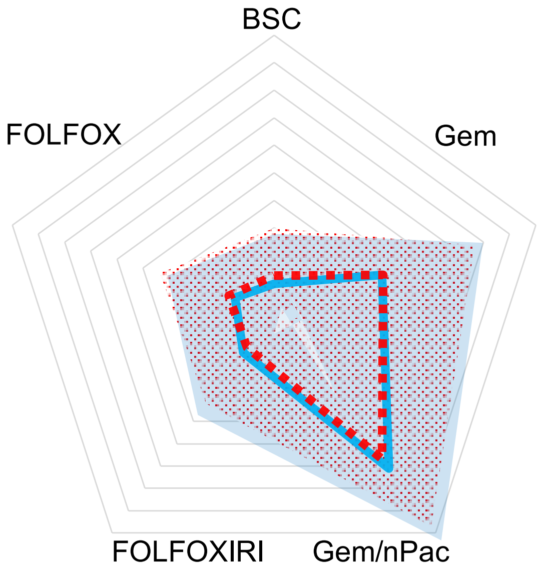

Figure 13: Radar plot for therapeutic recommendations for vignette G. The red and blue lines show the median, the light red and light blue areas the standard deviations of the treatment recommendations according “consultation / video” (actual age, video, comorbidities, medication, lab results provided; red graph) and according to the additional results of the “geriatric assessment” (including BI [Barthel Index], CIRS [Cumulative Illness Rating Scale], G8 [Geriatric 8], GDS [Geriatric Depression Scale], MMSE [Mini Mental Status Examination], MNA [Mini-Nutritional Assessment], TGUG [Timed Get Up and Go], QLQ-C30 [EORTC Quality of Life Questionnaire-C30]; blue graph); BSC = best supportive care only, FOLFOX = folinic acid, 5-fluorouracil and oxaliplatin, FOLFOXIRI = folinic acid, 5-fluorouracil, oxaliplatin and irinotecan, Gem = gemcitabine, Gem/nPac = gemcitabine/nab-paclitaxel, significant differences between recommendations marked with an asterisk, n= 15

Table S26: Comorbidities, prescriptions drugs and examinations of vignette G

**Comorbidities**	Essential hypertension, benign prostatic hyperplasia, status post colon cancer 17 years ago
**Prescription drugs**	Valsartan 160mg, Amlodipine 5mg, Moxonidine 0,3mg, hydrochlorothiazide 25mg, Spironolactone 50mg, Pangrol 25.000, Metamizole 500mg, Tilidin 50mg
**Examinations**	CT: Pancreatic head carcinoma with hepatic metastasis, status post hemicolectomy on the right.

Table S27: Lab results of vignette G

**Laboratory test**	**Result**	**Reference range**
Hemoglobin	10.9 mmol/L	7.4-10.7 mmol/L
Leucocytes	8.7 GPt/L	3.8-9.8 GPt/L
Thrombocytes	277 GPt/L	150-400 GPt/L
Creatinine	160 µmol/L	44-80 µmol/L
eGFR	36 mL/min/1.73	
Urea	5.2 mmol/L	3.5-7.2 mmol/L
ALAT	0.86 µmol/L	< 0.85 µmol/L
ASAT	0.62 µmol/L	< 0.85 µmol/L
Gamma-GT	9.8 µmol/L	< 1.19 µmol/L
LDH	5.6 µmol/L	2.3-3.37 µmol/L
Bilirubin (total)	13 µmol/L	< 21 µmol/L

Table S28: Results of geriatric assessment of vignette G

**Geriatric tool with reference range**	**Result**
Barthel Index (BI): activities of daily living<br>**100 points: completely independent**, 85-95: selectively in need of help, 35-80: in need of assistance, <30: care dependency	100/100 ■■■■■■■■■■
Cumulative Illness Rating Scale (CIRS): Comorbidities<br>0 = no comorbidities, 56 maximum points possible	12/56 ■■■■■■■■□□
G8: Geriatric screening tool for vulnerability<br>≤14 = abnormal screening	16/17 ■■■■■■■■■■
Geriatric Depression Scale (GDS)<br>**0-5: normal**, 6-10: mild depression, 11-15: severe depression	0/15 ■■■■■■■■■■
Mini-Mental-Status-Examination (MMSE)<br>**30-24: no / subtle cognitive deficits**, 23-18: mild cognitive deficits, ≤17 severe cognitive deficits	28/30 ■■■■■■■■■■
Mini Nutritional Assessment (MNA)<br>**24-30: normal nutritional status,** 17-23.5: risk for malnutrition, <17: malnutrition	29.5/30 ■■■■■■■■■■
Timed up and go test: Mobility test: get up from chair, walk 3m, return and sitz down, in seconds<br><10 seconds: no impairment, **10-19 seconds: less mobile**, 20-29 seconds: reduced mobility, >30 seconds: severely reduced mobility	14 ■■■■■■■■□□
Stair climb test (SCT) over 2 stories (7.7m) in seconds<br>(healthy individual: <30 seconds)	42 ■■■■■■■■□□
QLQ-C30: Summary Score<br>A high result means a **high level of functioning** and should be **regarded positively**	96/100 ■■■■■■■■■■
QLQ-C30: Functions<br>A high result means a **high level of functioning** and should be **regarded positively**	
Physical functioning	100/100 ■■■■■■■■■■
Role functioning	100/100 ■■■■■■■■■■
Emotional functioning	92/100 ■■■■■■■■■□
Cognitive functioning	100/100 ■■■■■■■■■■
Social functioning	100/100 ■■■■■■■■■■
QLQ-C30: Symptoms<br>A high result means a **high level of symptoms** and should be **regarded negatively**	
Fatigue	0/100 □□□□□□□□□□
Nausea and vomiting	0/100 □□□□□□□□□□
Pain	17/100 ■■□□□□□□□□
Dyspnea	0/100 □□□□□□□□□□
Insomnia	33/100 ■■■□□□□□□□
Loss of appetite	0/100 □□□□□□□□□□
Constipation	0/100 □□□□□□□□□□
Diarrhea	0/100 □□□□□□□□□□
Financial difficulties	0/100 □□□□□□□□□□

Vignette H

81 years old male patient with a pancreatic head carcinoma with liver metastasis (moderately differentiated, mucinous adenocarcinoma, G2).

Question: Palliative chemotherapy?

The following options were given:

1. Best supportive care only (BSC)
2. Gemcitabine (Gem)
3. Gemcitabine/nab-paclitaxel (Gem/nPac)
4. Folinic acid, 5-fluorouracil, oxaliplatin and irinotecan (FOLFOXIRI)
5. Folinic acid, 5-fluorouracil and oxaliplatin (FOLFOX)

Table S31 shows the descriptive statistics on the therapeutic recommendations followed by a graphical presentation. In the first graph, step 1 (50-years-old, no comorbidities, cross-sectional imaging, stage of disease) and step 2 (actual age, video, comorbidities, medication, lab results) are shown. In the second graph, step 2 and step 3 (results of GA) are shown.

Table S29: Recommended therapeutic regimes for vignette H

	**BSC**	**Gem**	**Gem/nPac**	**FOLFOXIRI**	**FOLFOX**
Guideline / tumor findings					
Mean ± SD	6 ± 12	20 ± 29	59 ± 35	86 ± 17	18 ± 20
Variance	148	815	1194	296	398
„Consultation / video“					
Mean ± SD	28 ± 31	75 ± 23	30 ± 33	6 ± 16	9 ± 17
Variance	966	510	1112	246	302
„Geriatric assessment“					
Mean ± SD	40 ± 33	67 ± 29	22 ± 30	5 ± 12	11 ± 20
Variance	1096	820	885	133	387

“Guideline / tumor findings“: 50-years-old patient without comorbidities, cross-sectional imaging, stage of disease provided; “consultation / video”: actual age, video, comorbidities, medication, lab results provided; “geriatric assessment”: BI [Barthel Index], CIRS [Cumulative Illness Rating Scale], G8 [Geriatric 8], GDS [Geriatric Depression Scale], MMSE [Mini Mental Status Examination], MNA [Mini-Nutritional Assessment], TGUG [Timed Get Up and Go], QLQ-C30 [EORTC Quality of Life Questionnaire-C30]). BSC = best supportive care only, FOLFOX = folinic acid, 5-fluorouracil and oxaliplatin, FOLFOXIRI = folinic acid, 5-fluorouracil, oxaliplatin and irinotecan, Gem = gemcitabine, Gem/nPac = gemcitabine/nab-paclitaxel, n= 21

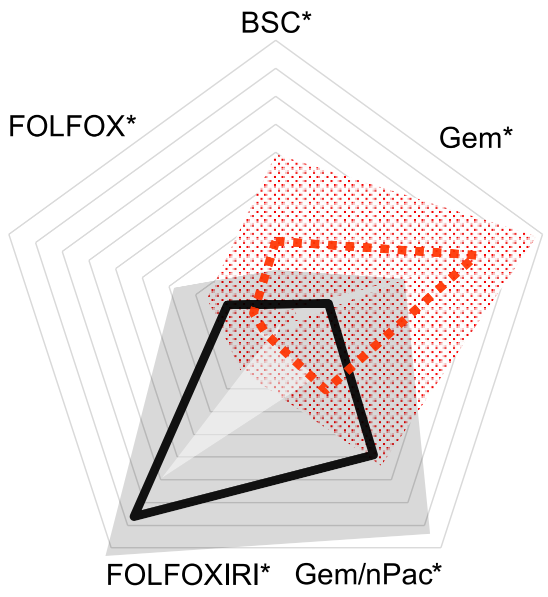

Figure 14: Radar plot for therapeutic recommendations for vignette H. The black and red lines show the median, the grey and light red areas the standard deviations of the treatment recommendations according to „guideline / tumor findings“ (“please assume 50-years-old patient without comorbidities”, cross-sectional imaging, stage of disease provided; black graph) and according to the “consultation / video” (actual age, video, comorbidities, medication, lab results provided; red graph); BSC = best supportive care only, FOLFOX = folinic acid, 5-fluorouracil and oxaliplatin, FOLFOXIRI = folinic acid, 5-fluorouracil, oxaliplatin and irinotecan, Gem = gemcitabine, Gem/nPac = gemcitabine/nab-paclitaxel, significant differences between recommendations marked with an asterisk, n= 21

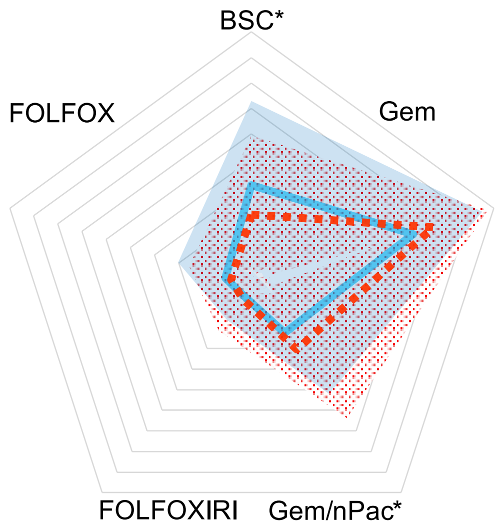

Figure 15: Radar plot for therapeutic recommendations for vignette H. The red and blue lines show the median, the light red and light blue areas the standard deviations of the treatment recommendations according “consultation / video” (actual age, video, comorbidities, medication, lab results provided; red graph) and according to the additional results of the “geriatric assessment” (including BI [Barthel Index], CIRS [Cumulative Illness Rating Scale], G8 [Geriatric 8], GDS [Geriatric Depression Scale], MMSE [Mini Mental Status Examination], MNA [Mini-Nutritional Assessment], TGUG [Timed Get Up and Go], QLQ-C30 [EORTC Quality of Life Questionnaire-C30]; blue graph); BSC = best supportive care only; FOLFOX = folinic acid, 5-fluorouracil and oxaliplatin, FOLFOXIRI = folinic acid, 5-fluorouracil, oxaliplatin and irinotecan, Gem = gemcitabine, Gem/nPac = gemcitabine/nab-paclitaxel, significant differences between recommendations marked with an asterisk, n= 21

Table S30: Comorbidities, prescriptions drugs and examinations of vignette H

**Comorbidities**	Atrial fibrillation, mitral regurgitation II°, tricuspid regurgitation I°, essential hypertension, hyperlipoproteinemia, benign prostatic hyperplasia, presbycusis
**Prescription drugs**	Aspirin 100mg, Ramipril 5mg, Metoprolol 47.5mg, Rivaroxaban 20mg, Atorvastatin 40mg.
**Examinations**	CT: Pancreatic head carcinoma. Liver metastases in both lobes of the liver. Stenting for extra- and intrahepatic cholestasis.

Table S31: Lab results of vignette H

**Laboratory test**	**Result**	**Reference range**
Hemoglobin	9.1 mmol/L	7.4-10.7 mmol/L
Leucocytes	5.8 GPt/L	3.8-9.8 GPt/L
Thrombocytes	224 GPt/L	150-400 GPt/L
Creatinine	89 µmol/L	44-80 µmol/L
eGFR	77 mL/min/1.73	
Urea	5.9 mmol/L	3.5-7.2 mmol/L
ALAT	0.52 µmol/L	< 0.85 µmol/L
ASAT	0.68 µmol/L	< 0.85 µmol/L
Gamma-GT	13.29 µmol/L	< 1.19 µmol/L
LDH	6.31 µmol/L	2.3-3.37 µmol/L
Bilirubin (total)	10.24 µmol/L	< 21 µmol/L

Table S32: Results of geriatric assessment of vignette H

Geriatric tool with reference range	Result
Barthel Index (BI): activities of daily living<br>100 points: completely independent, 85-95: selectively in need of help, **35-80: in need of assistance**, <30: care dependency	60/100 ■■■■■■□□□□
Cumulative Illness Rating Scale (CIRS): Comorbidities<br>0 = no comorbidities, 56 maximum points possible	17/56 ■■■■■■■□□□
G8: Geriatric screening tool for vulnerability<br>**≤14 = abnormal screening**	8/17 ■■■■■■□□□□
Geriatric Depression Scale (GDS)<br>**0-5: normal**, 6-10: mild depression, 11-15: severe depression	3/15 ■■■■■■■■■■
Mini-Mental-Status-Examination (MMSE)<br>**30-24: no / subtle cognitive deficits**, 23-18: mild cognitive deficits, ≤17 severe cognitive deficits	27/30 ■■■■■■■■■■
Mini Nutritional Assessment (MNA)<br>24-30: normal nutritional status, **17-23.5: risk for malnutrition**, <17: malnutrition	19/30 ■■■□□□□□□□
Timed up and go test: Mobility test: get up from chair, walk 3m, return and sitz down, in seconds<br><10 seconds: no impairment, 10-19 seconds: less mobile, **20-29 seconds: reduced mobility**, >30 seconds: severely reduced mobility	26 ■■■□□□□□□□
Stair climb test (SCT) over 2 stories (7.7m) in seconds<br>(healthy individual: <30 seconds)	n.a.
QLQ-C30: Summary Score<br>A high result means a **high level of functioning** and should be **regarded positively**	46/100 ■■■■■□□□□□
QLQ-C30: Functions<br>A high result means a **high level of functioning** and should be **regarded positively**	
Physical functioning	20/100 ■■□□□□□□□□
Role functioning	0/100 □□□□□□□□□□
Emotional functioning	42/100 ■■■■□□□□□□
Cognitive functioning	83/100 ■■■■■■■■□□
Social functioning	0/100 □□□□□□□□□□
QLQ-C30: Symptoms<br>A high result means a **high level of symptoms** and should be **regarded negatively**	
Fatigue	78/100 ■■■■■■■■□□
Nausea and vomiting	33/100 ■■■□□□□□□□
Pain	0/100 □□□□□□□□□□
Dyspnea	67/100 ■■■■■■■□□□
Insomnia	67/100 ■■■■■■■□□□
Loss of appetite	67/100 ■■■■■■■□□□
Constipation	33/100 ■■■□□□□□□□
Diarrhea	0/100 □□□□□□□□□□
Financial difficulties	0/100 □□□□□□□□□□

Vignette I

78-year-old male patient with a coecum carcinoma (pT3b pN2b (7/36) L1 V0 R0, mucinous adenocarcinoma, low grade MSI-H, B-RAF: wild type, G2).

Question: Adjuvant chemotherapy?

The following options were given:

- No adjuvant therapy (no adj. th.)
- Capecitabine (Cape)
- Infusional 5-fluorouracil (5-FU)
- Oxaliplatin (Ox)

Table S35 shows the descriptive statistics on the therapeutic recommendations followed by a graphical presentation. In the first graph, step 1 (50-years-old, no comorbidities, cross-sectional imaging, stage of disease) and step 2 (actual age, video, comorbidities, medication, lab results) are shown. In the second graph, step 2 and step 3 (results of GA) are shown.

Table S33: Recommended therapeutic regimes for vignette I

	No adj. th.	Cape	5-FU	Ox
Guideline / tumor findings				
Mean ± SD	16 ± 31	36 ± 35	29 ± 34	88 ± 19
Variance	957	1251	1140	348
„Consultation / video“				
Mean ± SD	19 ± 33	64 ± 32	38 ± 41	54 ± 33
Variance	1099	996	1654	1069
„Geriatric assessment“				
Mean ± SD	12 ± 27	66 ± 32	39 ± 41	54 ± 39
Variance	731	995	1659	1517

“Guideline / tumor findings“: 50-years-old patient without comorbidities, cross-sectional imaging, stage of disease provided; “consultation / video”: actual age, video, comorbidities, medication, lab results provided; “geriatric assessment”: BI [Barthel Index], CIRS [Cumulative Illness Rating Scale], G8 [Geriatric 8], GDS [Geriatric Depression Scale], MMSE [Mini Mental Status Examination], MNA [Mini-Nutritional Assessment], TGUG [Timed Get Up and Go], QLQ-C30 [EORTC Quality of Life Questionnaire-C30]). 5-FU = infusional 5-fluorouracil, Cape = capecitabine, No adj. th. = no adjuvant therapy, Ox = oxaliplatin, n= 11

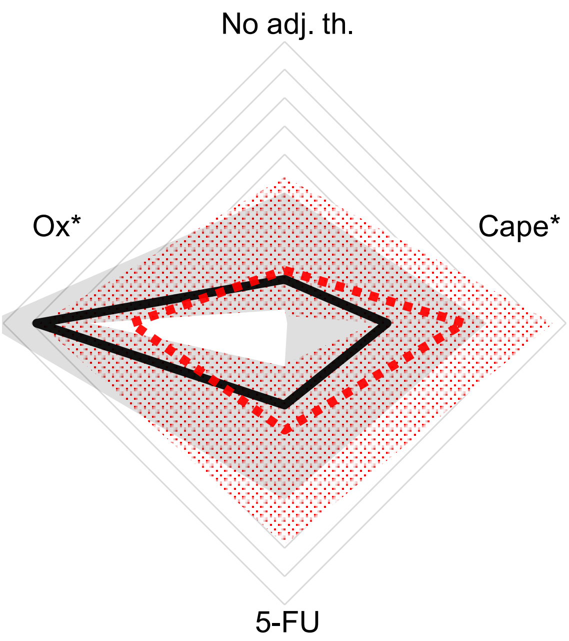

Figure 16: Radar plot for therapeutic recommendations for vignette I. The black and red lines show the median, the grey and light red areas the standard deviations of the treatment recommendations according to „guideline / tumor findings“ (“please assume 50-years-old patient without comorbidities”, cross-sectional imaging, stage of disease provided; black graph) and according to the “consultation / video” (actual age, video, comorbidities, medication, lab results provided; red graph); 5-FU = infusional 5-fluorouracil, Cape = capecitabine, No adj. th. = no adjuvant therapy, Ox = oxaliplatin, significant differences between recommendations marked with an asterisk, n= 11

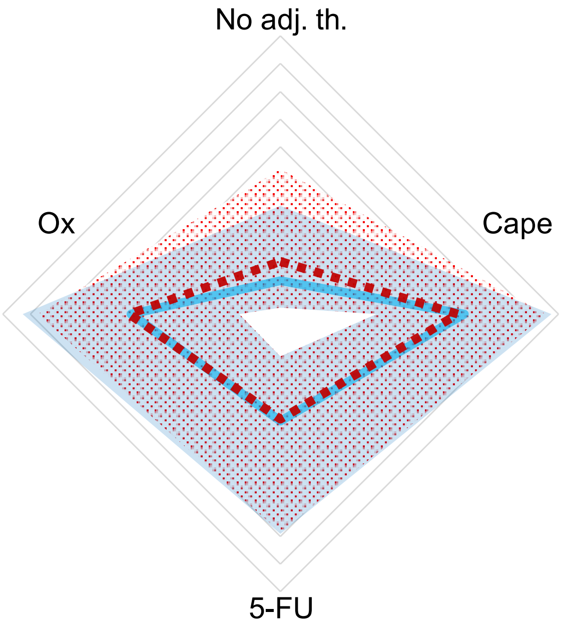

Figure 17: Radar plot for therapeutic recommendations for vignette I. The red and blue lines show the median, the light red and light blue areas the standard deviations of the treatment recommendations according “consultation / video” (actual age, video, comorbidities, medication, lab results provided; red graph) and according to the additional results of the “geriatric assessment” (including BI [Barthel Index], CIRS [Cumulative Illness Rating Scale], G8 [Geriatric 8], GDS [Geriatric Depression Scale], MMSE [Mini Mental Status Examination], MNA [Mini-Nutritional Assessment], TGUG [Timed Get Up and Go], QLQ-C30 [EORTC Quality of Life Questionnaire-C30]; blue graph); 5-FU = infusional 5-fluorouracil, Cape = capecitabine, No adj. th. = no adjuvant therapy, Ox = oxaliplatin, significant differences between recommendations marked with an asterisk, n= 11

Table S34: Comorbidities, prescriptions drugs and examinations of vignette I

**Comorbidities**	Essential hypertension, benign prostatic hyperplasia, macular degeneration, kidney stone disease, status post double J stents right, status post umbilical hernia. Allergies: Iodinated contrast media
**Prescription drugs**	Pantoprazole 20mg, Telmisartan 80mg, Amlodipine 5mg, Metamizole up to 4g/d
**Examinations**	Preoperative CT: No distant metastases.
	Colonoscopy: Ulcerating tumor in C. asc. / coecum

Table S35: Lab results of vignette I

**Laboratory test**	**Result**	**Reference range**
Hemoglobin	7.3 mmol/L	7.4-10.7 mmol/L
Leucocytes	7.31 GPt/L	3.8-9.8 GPt/L
Thrombocytes	285 GPt/L	150-400 GPt/L
Creatinine	78 µmol/L	44-80 µmol/L
eGFR	82 mL/min/1.73	
Urea	3.7 mmol/L	3.5-7.2 mmol/L
ALAT	0.25 µmol/L	< 0.85 µmol/L
ASAT	0.24 µmol/L	< 0.85 µmol/L
Gamma-GT	0.53 µmol/L	< 1.19 µmol/L
LDH	2.89 µmol/L	2.3-3.37 µmol/L
Bilirubin (total)	5.5 µmol/L	< 21 µmol/L

Table S36: Results of geriatric assessment of vignette I

Geriatric tool with reference range	Result
Barthel Index (BI): activities of daily living<br>**100 points: completely independent**, 85-95: selectively in need of help, 35-80: in need of assistance, <30: care dependency	100/100 ■■■■■■■■■■
Cumulative Illness Rating Scale (CIRS): Comorbidities<br>0 = no comorbidities, 56 maximum points possible	15/56 ■■■■■■■□□□
G8: Geriatric screening tool for vulnerability<br>**≤14 = abnormal screening**	13/17 ■■■■■■■■■□
Geriatric Depression Scale (GDS)<br>**0-5: normal**, 6-10: mild depression, 11-15: severe depression	0/15 ■■■■■■■■■■
Mini-Mental-Status-Examination (MMSE)<br>**30-24: no / subtle cognitive deficits**, 23-18: mild cognitive deficits, ≤17 severe cognitive deficits	30/30 ■■■■■■■■■■
Mini Nutritional Assessment (MNA)<br>24-30: normal nutritional status, **17-23.5: risk for malnutrition,** <17: malnutrition	23.5/30 ■■■■■■■■■□
Timed up and go test: Mobility test: get up from chair, walk 3m, return and sitz down, in seconds<br>**<10 seconds: no impairment**, 10-19 seconds: less mobile, 20-29 seconds: reduced mobility, >30 seconds: severely reduced mobility	8 ■■■■■■■■■■
Stair climb test (SCT) over 2 stories (7.7m) in seconds<br>(healthy individual: <30 seconds)	41 ■■■■■■■■□□
QLQ-C30: Summary Score<br>A high result means a **high level of functioning** and should be **regarded positively**	94/100 ■■■■■■■■■□
QLQ-C30: Functions<br>A high result means a **high level of functioning** and should be **regarded positively**	
Physical functioning	93/100 ■■■■■■■■■□
Role functioning	100/100 ■■■■■■■■■■
Emotional functioning	83/100 ■■■■■■■■□□
Cognitive functioning	100/100 ■■■■■■■■■■
Social functioning	100/100 ■■■■■■■■■■
QLQ-C30: Symptoms<br>A high result means a **high level of symptoms** and should be **regarded negatively**	
Fatigue	0/100 □□□□□□□□□□
Nausea and vomiting	0/100 □□□□□□□□□□
Pain	17/100 ■■□□□□□□□□
Dyspnea	0/100 □□□□□□□□□□
Insomnia	0/100 □□□□□□□□□□
Loss of appetite	33/100 ■■■□□□□□□□
Constipation	0/100 □□□□□□□□□□
Diarrhea	0/100 □□□□□□□□□□
Financial difficulties	0/100 □□□□□□□□□□

Vignette K

83 years old male patient with rectal cancer (ypT2 ypN2b (8/13) L1 V1 Pn0 RX M0, moderately to poorly differentiated adenocarcinoma, MSS).

Question: Adjuvant chemotherapy?

The following options were given:

1. No adjuvant therapy (no adj. th)
2. Capecitabine (Cape)
3. Infusional 5-fluorouracil (5-FU)
4. Oxaliplatin based (Ox)

Table S39 shows the descriptive statistics on the therapeutic recommendations followed by a graphical presentation. In the first graph, step 1 (50-years-old, no comorbidities, cross-sectional imaging, stage of disease) and step 2 (actual age, video, comorbidities, medication, lab results) are shown. In the second graph, step 2 and step 3 (results of GA) are shown.

Table S37: Recommended therapeutic regimes for vignette K

	No adj. th	Cape	5-FU	Ox
Guideline / tumor findings				
Mean ± SD	7 ± 15	50 ± 37	36 ± 41	74 ± 33
Variance	213	1336	1705	1070
„Consultation / video“				
Mean ± SD	36 ± 38	62 ± 37	19 ± 27	12 ± 23
Variance	1426	1390	744	550
„Geriatric assessment“				
Mean ± SD	38 ± 38	57 ± 36	23 ± 28	14 ± 30
Variance	1418	1271	808	877

“Guideline / tumor findings“: 50-years-old patient without comorbidities, cross-sectional imaging, stage of disease provided; “consultation / video”: actual age, video, comorbidities, medication, lab results provided; “geriatric assessment”: BI [Barthel Index], CIRS [Cumulative Illness Rating Scale], G8 [Geriatric 8], GDS [Geriatric Depression Scale], MMSE [Mini Mental Status Examination], MNA [Mini-Nutritional Assessment], TGUG [Timed Get Up and Go], QLQ-C30 [EORTC Quality of Life Questionnaire-C30]). 5-FU = infusional 5-fluorouracil, Cape = capecitabine, No adj. th = no adjuvant therapy, Ox = oxaliplatin based, n= 24.

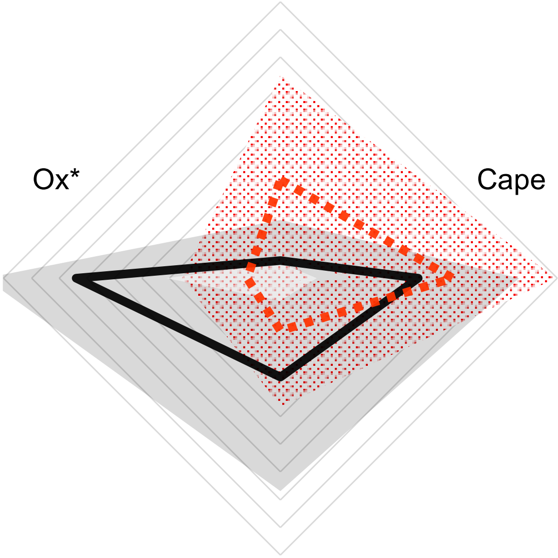

Figure 18: Radar plot for therapeutic recommendations for vignette K. The black and red lines show the median, the grey and light red areas the standard deviations of the treatment recommendations according to „guideline / tumor findings“ (“please assume 50-years-old patient without comorbidities”, cross-sectional imaging, stage of disease provided; black graph) and according to the “consultation / video” (actual age, video, comorbidities, medication, lab results provided; red graph); 5-FU = infusional 5-fluorouracil, Cape = capecitabine, No adj. th = no adjuvant therapy, Ox = oxaliplatin based, significant differences between recommendations marked with an asterisk, n=24.

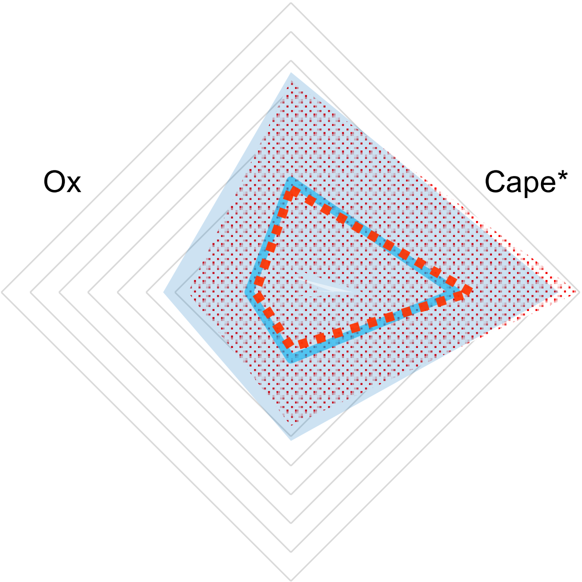

Figure 19: Radar plot for therapeutic recommendations for vignette K. The red and blue lines show the median, the light red and light blue areas the standard deviations of the treatment recommendations according “consultation / video” (actual age, video, comorbidities, medication, lab results provided; red graph) and according to the additional results of the “geriatric assessment” (including BI [Barthel Index], CIRS [Cumulative Illness Rating Scale], G8 [Geriatric 8], GDS [Geriatric Depression Scale], MMSE [Mini Mental Status Examination], MNA [Mini-Nutritional Assessment], TGUG [Timed Get Up and Go], QLQ-C30 [EORTC Quality of Life Questionnaire-C30]; blue graph); 5-FU = infusional 5-fluorouracil, Cape = capecitabine, No adj. th = no adjuvant therapy, Ox = oxaliplatin based, significant differences between recommendations marked with an asterisk, n= 24.

Table S38: Comorbidities, prescriptions drugs and examinations of vignette K

**Comorbidities**	Essential hypertension, moderate aortic stenosis, type 2 diabetes, prostatic cancer (1 year ago, not the oncological focus), status post transurethral resection of the prostate (18 years ago), status post kyphoplasty L5 (2 years ago)
**Prescription drugs**	Ramipril 5mg, hydrochlorothiazide 25mg
**Examinations**	Preoperative CT: No evidence of infiltration of the mesorectal fascia. Highly suspected lymph node infiltration mesorectally on the left and in the course of the superior rectal artery. No inguinal lymph node metastasis. No distant metastasis.
	Colonoscopy: 8 cm from ano: semicircular exulcerated tumor. Sigmoid and colon: multiple non-irritant diverticula without obstruction of passage.

Table S39: Lab results of vignette K

**Laboratory test**	**Result**	**Reference range**
Hemoglobin	6.0 mmol/L	7.4-10.7 mmol/L
Leucocytes	7.82 GPt/L	3.8-9.8 GPt/L
Thrombocytes	240 GPt/L	150-400 GPt/L
Creatinine	85 µmol/L	44-80 µmol/L
eGFR	73 mL/min/1.73	
Urea	5.4 mmol/L	3.5-7.2 mmol/L
ALAT	0.43 µmol/L	< 0.85 µmol/L
ASAT	0.69 µmol/L	< 0.85 µmol/L
Gamma-GT	0.28 µmol/L	< 1.19 µmol/L
LDH	3.61 µmol/L	2.3-3.37 µmol/L
Bilirubin (total)	4.5 µmol/L	< 21 µmol/L

Table S40: Results of geriatric assessment of vignette K

Geriatric tool with reference range	Result
Barthel Index (BI): activities of daily living<br>100 points: completely independent, 85-95: selectively in need of help, **35-80: in need of assistance**, <30: care dependency	75/100 ■■■■■■■□□
Cumulative Illness Rating Scale (CIRS): Comorbidities<br>0 = no comorbidities, 56 maximum points possible	15/56 ■■■■■■□□□
G8: Geriatric screening tool for vulnerability<br>**≤14 = abnormal screening**	10/17 ■■■■■■□□□
Geriatric Depression Scale (GDS)<br>**0-5: normal**, 6-10: mild depression, 11-15: severe depression	3/15 ■■■■■■■■■■
Mini-Mental-Status-Examination (MMSE)<br>**30-24: no / subtle cognitive deficits**, 23-18: mild cognitive deficits, ≤17 severe cognitive deficits	24/30 ■■■■■■■□□
Mini Nutritional Assessment (MNA)<br>24-30: normal nutritional status, **17-23.5: risk for malnutrition**, <17: malnutrition	21/30 ■■■■■■□□□
Timed up and go test: Mobility test: get up from chair, walk 3m, return and sitz down, in seconds<br><10 seconds: no impairment, **10-19 seconds: less mobile**, 20-29 seconds: reduced mobility, >30 seconds: severely reduced mobility	14 ■■■■■■■□□
Stair climb test (SCT) over 2 stories (7.7m) in seconds<br>(healthy individual: <30 seconds)	41 ■■■■■■■□□
QLQ-C30: Summary Score<br>A high result means a **high level of functioning** and should be **regarded positively**	73/100 ■■■■■■□□□
QLQ-C30: Functions<br>A high result means a **high level of functioning** and should be **regarded positively**	
Physical functioning	27/100 ■■■□□□□□□□
Role functioning	67/100 ■■■■■■■□□□
Emotional functioning	83/100 ■■■■■■■■□□
Cognitive functioning	83/100 ■■■■■■■■□□
Social functioning	33/100 ■■■□□□□□□□
QLQ-C30: Symptoms<br>A high result means a **high level of symptoms** and should be **regarded negatively**	
Fatigue	33/100 ■■■□□□□□□□
Nausea and vomiting	50/100 ■■■■■□□□□□
Pain	0/100 □□□□□□□□□□
Dyspnea	0/100 □□□□□□□□□□
Insomnia	67/100 ■■■■■■■□□□
Loss of appetite	0/100 □□□□□□□□□□
Constipation	0/100 □□□□□□□□□□
Diarrhea	0/100 □□□□□□□□□□
Financial difficulties	0/100 □□□□□□□□□□
